# Idiosyncratic responses to biotic and environmental filters in wood‐inhabiting fungal communities

**DOI:** 10.1002/ecy.70013

**Published:** 2025-02-12

**Authors:** Sonja Saine, Reijo Penttilä, Tadashi Fukami, Brendan Furneaux, Tuija Hytönen, Otto Miettinen, Norman Monkhouse, Raisa Mäkipää, Jorma Pennanen, Evgeny V. Zakharov, Otso Ovaskainen, Nerea Abrego

**Affiliations:** ^1^ Department of Agricultural Sciences University of Helsinki Helsinki Finland; ^2^ Natural Resources Institute Finland (Luke) Helsinki Finland; ^3^ Department of Biology and Earth System Science Stanford University Stanford California USA; ^4^ Department of Biological and Environmental Science University of Jyväskylä Jyväskylä Finland; ^5^ Finnish Museum of Natural History University of Helsinki Helsinki Finland; ^6^ The Canadian Centre for DNA Barcoding, Centre for Biodiversity Genomics University of Guelph Guelph Ontario Canada; ^7^ Present address: Organismal and Evolutionary Biology Research Programme, Faculty of Biological and Environmental Sciences University of Helsinki Helsinki Finland

**Keywords:** colonization, community assembly, environmental conditions, field experiment, interspecific interactions, metacommunity, wood‐inhabiting fungi

## Abstract

Quantification of different processes affecting the assembly of ecological communities remains challenging, especially in species‐rich communities. While the role of environmental filtering has generally been well established, fewer studies have experimentally shown how other ecological assembly processes, such as biotic filtering, structure species‐rich communities. Here, we studied the relative roles of biotic and environmental filtering in the colonization of wood‐inhabiting fungi, a species‐rich, highly interactive, and environment‐sensitive group of species. We conducted a field experiment where we simulated colonization with inoculations of nine fungal species in habitat patches (i.e., logs) with varying biotic and abiotic conditions. We characterized the local resident communities before the inoculations and the colonization success of the inoculated species after one and two years using DNA metabarcoding. We asked what determined the colonization success of the inoculated species by comparing the predictive performance of alternative models. These models included either only abiotic environmental predictors (i.e., physical log properties) or additionally different aspects of the resident fungal communities (i.e., resident fungal species richness, community composition, and DNA amount) as biotic predictors. While all nine species successfully colonized the logs, the rate of success and the factors explaining their colonization success varied among species. The colonization success of four of the inoculated species was explained mostly by the abiotic environmental variables, while the colonization success of three species was additionally explained by the resident communities. The influential biotic predictors varied from the presence of individual species to the collective presence of multiple species. Finally, for two of the inoculated species, all the models showed poor predictive performance. Our results indicate how environmental and biotic filtering may jointly structure species‐rich communities. Overall, the results show that species vary idiosyncratically in their response to biotic and environmental factors, highlighting the need to consider the complexity of species‐level responses when predicting community‐level changes.

## INTRODUCTION

Quantification of different processes affecting ecological community assembly remains challenging, especially in species‐rich communities. In the literature, the importance of environmental filtering has been emphasized (e.g., Glassman et al., 2017; Laliberté et al., 2014; Lebrija‐Trejos et al., 2010). The effects of environmental filtering on community composition are generally inferred by relating environmental variation in the data to variation in metrics summarizing community‐wide patterns across all species. Yet, as species may respond differently to their environment, disentangling the mechanisms by which assembly processes structure the communities may also require a species‐level perspective (Baselga & Araújo, 2009). Biotic filtering, on the other hand, is typically inferred in species‐rich communities by applying statistical methods that allow separating the species co‐occurrences that cannot be explained by the measured environmental factors (e.g., Ovaskainen et al., 2010; Pollock et al., 2014). When applied to non‐manipulative data, however, these kinds of analyses do not yield conclusive proof of biotic filtering, since multiple processes can lead to similar patterns in the data (Logue et al., 2011; Münkemüller et al., 2020; Ovaskainen et al., 2019). Conducting manipulative experiments designed to capture the underlying assembly processes both at the community and species level is thus needed to gain a mechanistic understanding of the community dynamics.

Wood‐inhabiting fungi represent a convenient system to experimentally study community assembly because deadwood units (e.g., logs and snags) can be viewed as spatially and temporally well‐defined habitat patches (Abrego, 2022). These species form highly interactive and environment‐sensitive communities that simultaneously alter their habitats while decomposing deadwood. Environmental filtering is known to strongly influence wood‐inhabiting fungal communities through changes in resource quality and availability (e.g., Bässler et al., 2010; Hottola et al., 2009). Biotic filtering shapes wood‐inhabiting fungal communities through competitive and facilitative interactions (Woodward & Boddy, 2008). Laboratory experiments have revealed direct and indirect interactions, including competition for nutrients and space, growth inhibition or stimulation through metabolite secretion or modification of the substrate, and parasitism (e.g., Boddy, 2000; Heilmann‐Clausen & Boddy, 2005; Hiscox et al., 2018). Priority effects, which manifest as a joint outcome of direct and indirect interactions, influence succession in wood‐inhabiting fungal communities (Fukami et al., 2010; Hiscox et al., 2015).

In this study, we carried out a field experiment to investigate the relative roles of environmental and biotic filtering in determining the colonization success of different wood‐inhabiting fungal species. We simulated colonization by introducing fungal species via inoculations in fresh naturally fallen and artificially felled Norway spruce logs representing different abiotic and biotic conditions. Abiotically, natural and felled logs differ in their physiochemical properties due to different mortality factors (Stokland & Siitonen, 2012), which is further reflected in biotic differences as these logs can host distinct fungal communities (e.g., Pasanen et al., 2018; Saine, Penttilä, Furneaux, et al., 2024). We used DNA‐based sampling to characterize the resident fungal communities before the inoculations and to monitor the colonization success of the inoculated fungal species after one and two years. To address the relative effects of environmental and biotic filtering on the colonization success of each inoculated species, we fitted alternative statistical models. The alternative models included either only environmental predictors, describing the physical and chemical properties of the logs, or also biotic predictors, describing different aspects of the resident fungal communities, such as fungal species richness, community composition, and DNA amount. We then compared the predictive performance of the models to assess whether colonization success could be predicted mostly by the abiotic environmental conditions or additionally by the resident fungal community (representative of environmental and biotic filtering, respectively). Given the large taxonomic and functional diversity of wood‐inhabiting fungi within individual logs (Lustenhouwer et al., 2020; Ottosson et al., 2015), we expected that their coexistence is driven by a high degree of biotic and abiotic niche partitioning across species. This, in turn, should be reflected in a large degree of idiosyncratic responses to different biotic and abiotic predictors. Namely, we hypothesized that the relative roles of environmental and biotic filtering would vary among species. In addition, given the many types of interactions that have been reported for wood‐inhabiting fungi, including both direct and indirect, as well as positive and negative interactions, we expected to find interspecific variation in the type of biotic predictors of the colonization success.

## METHODS

### Study sites and experimental design

**FIGURE 1:**
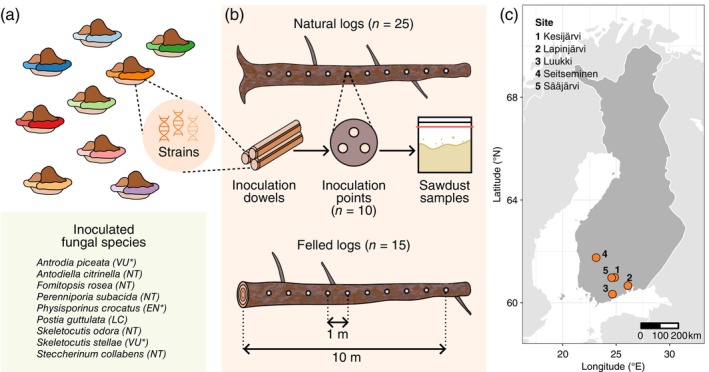
Illustration of the experiment: (a) the inoculated fungal species, (b) the study design, and (c) map of the study sites in Finland. (a) We inoculated nine wood‐inhabiting fungal species that each were represented by two to six strains. Red‐list statuses shown after the species names follow the 2019 Red List of Finnish Species (Kotiranta et al., 2019; EN, endangered; LC, least concern; NT, near threatened; VU, vulnerable). Threatened species are marked with an asterisk. (b) Each species was inoculated in 25 natural and 15 felled Norway spruce logs across the five study sites, totaling inoculations of 225 natural and 135 felled logs for the whole experiment. We conducted the inoculations with dowels containing mycelia of the target strains. For every log, we inoculated one species in 10 inoculation points. At each inoculation point, we drilled three holes and introduced six dowels of a randomly selected strain. We collected sawdust samples at these inoculation points three times: once before as well as one and two years after the inoculations. All illustrations were created by the authors.

The experimental design was the same as for Saine, Penttilä, Furneaux, et al. (2024). We carried out the experiment at five forest sites in Finland (Figure 1c). All sites were dominated by Norway spruce (*Picea abies* [L.] Karst) in middle‐aged or mature stands characterized by a natural‐like forest structure with high quantities of deadwood (Appendix S1: Table S1). The size of the experimental areas varied from 2 to 5 ha (Appendix S1: Table S1), depending on the availability of spruce logs and living trees filling the following selection criteria. Selected spruce logs and living spruces had a dbh ≥20 cm (measured 1.3 m from the base). Selected logs were preferably in decay stage 1 on a scale from 1 to 5 (Renvall, 1995) but also in decay stage 2 if logs in decay stage 1 were not available. All felled logs were in decay stage 1, while 65% of natural logs were in decay stage 1 and 35% in decay stage 2. To create the felled logs, we cut the selected living spruces at the base with a chain saw in April–May 2019. The selected trees were healthy without visible signs of infection. The selected natural logs were either uprooted or broken (50% and 50% of all natural logs, respectively). Each site included 55 natural and 37 felled logs. A site‐level summary of the characteristics of the study logs is presented in Appendix S1: Table S2.

### Fungal inoculations

For the inoculations, we chose nine wood‐inhabiting fungal species (Figure 1a). All the inoculated species belong to the order Polyporales and use spruce as their primary host (Niemelä, 2016). All but one of them are currently red‐listed in Finland (Kotiranta et al., 2019; Figure 1a). Three of the species are brown rot fungi and six are white rot fungi. See Appendix S1: Section S2.1 for more information on the distribution and status of the target species. In addition to utilizing the University of Helsinki culture collections, we collected source material for new fungal cultures by sampling target species' fruit bodies in the field in August–November 2018 (Appendix S1: Section S2.2). To ensure genetic variation for inoculated species, each species was represented by individuals from 2 to 6 different locations in Finland (Appendix S1: Table S4 and Figure S1).

The general workflow for strain isolation, cultivation, and inoculation followed Abrego et al. (2016), with slight modifications. In the laboratory, the strains were grown at room temperature, and their growth was visually checked on a regular basis. If contamination was detected, uncontaminated parts of mycelia were transferred to new agar plates. We allowed mycelia to fill the agar plates, taking 11–74 days (Appendix S1: Table S4), and Sanger‐sequenced every strain to confirm their identification (Appendix S1: Section S2.2). Inoculation dowels were prepared by Kääpä Biotech Oy (Karjalohja, Finland). The mycelia of each strain in the agar plates were cut into pieces and placed in plastic growing bags filled with oat grains. After one to two weeks, 50 × 10 mm sterilized wooden dowels made of industrial spruce timber (Helsingin Erikoishöyläys Oy) were inserted into the bags. Mycelia colonized the dowels in one to two months depending on the target species.

To conduct the inoculations in the field, the colonized dowels were inserted at 10 inoculation points within each log. The inoculation points were located 1 m apart on the same side of the log, starting 1 m from the base (or root collar for uprooted natural logs) and extending up to 10 m (Figure 1b). At each inoculation point, we drilled three holes in a triangular shape (ca. 3 cm apart; Figure 1b), and within each hole, we inserted two inoculation dowels one after another. We drilled the holes perpendicularly towards the log center using a cordless drill (Makita, model DDF481) with a 11 x 105 mm wood drill bit. We sterilized the drill bit after each log by soaking it in 5% sodium hypochlorite (NaClO) for at least 3 min, then rinsing it in water followed by ethanol. To prevent contamination, we covered the inoculation holes with gardening wax (Neko, Oy Neko Ab, Finland).

We inoculated each log with one randomly chosen species and each inoculation point with one randomly selected strain. All nine target species were inoculated in five natural logs and three felled logs at each of the five sites, for a total of 40 logs per species (hereafter, the target logs for that species). In addition, we inoculated 10 natural and 10 felled logs per site with sterilized dowels without mycelia as controls. In total, we inoculated 460 logs. Before the inoculations, we visually checked that the target species' fruit bodies did not occur on the study logs. Additionally, within and in the immediate surroundings of each study site, we carried out an 8‐h survey for the target species by two fungal experts to estimate their natural occurrence levels. The surveys revealed that *Fomitopsis rosea* naturally occurred in the surroundings of three sites (on nine non‐study logs) and that *Antrodiella citrinella* occurred in the surroundings of one site (on one non‐study log). As the natural occurrences were few and not exactly within the experimental areas, we proceeded with the inoculations as planned.

### DNA‐based survey of fungal communities

We surveyed the fungal communities through DNA‐based surveys of sawdust samples. For each study log, we collected sawdust samples three times: once before the inoculations to characterize the resident fungal communities and twice after the inoculations to monitor the colonization success of the inoculated species after one and two years. We collected the first set of sawdust samples in August–October 2019 at the same time as the inoculations, the second in August–September 2020, and the third in August–September 2021. In the first year, the sawdust samples were obtained by collecting the sawdust resulting from the drilling for the inoculation dowels as described above. The next two sets of sawdust samples were collected by drilling ca. 2 cm away from the inoculation point. Before drilling, we removed the bark to avoid sampling fungal DNA present in some other form than wood‐inhabiting mycelia. For the subsequent DNA analyses, samples from different inoculation points were pooled, resulting in one data point per log per year, totaling 1380 samples.

### Sample preprocessing, sequencing, and bioinformatic analyses

The workflow for sample preprocessing, DNA extraction, sequencing, and bioinformatic analyses was the same as described in Saine, Penttilä, Furneaux, et al. (2024). See Appendix S1: Section S3 for more details on each step. After collection in the field, all samples were stored at −20°C. Before DNA analyses, the samples were freeze‐dried and pulverized. For DNA sequencing, we targeted part of the ribosomal internal transcribed spacer (ITS) region and applied a spike‐in approach to generate quantitative estimates of DNA amount, more specifically the weight of the ITS that can be amplified by our protocol using polymerace chain reaction (PCR) (see Ovaskainen et al., 2020). We note that while such estimated DNA amount is influenced by PCR bias and variation in ITS sequence length, we consider it to capture biologically meaningful variation. This is evidenced by, for example, the earlier research finding where similarly estimated DNA amount showed predictable spatial and seasonal variation (Abrego et al., 2024). We performed the bioinformatic analyses using a development version of the OptimOTU pipeline that was implemented in R (version 4.2.2; R Core Team, 2022). The processing steps included raw read filtering and trimming using Cutadapt (version 4.2; Martin, 2011) and DADA2 (version 1.26; Callahan et al., 2016), followed by denoising to form amplicon sequence variants (ASVs), and ASV filtering and trimming with DADA2. Then, we performed probabilistic taxonomic identification of the ASVs with Protax‐fungi (Abarenkov et al., 2018) to form taxonomically guided operational taxonomic units (OTUs). During this final clustering step, many ASVs are merged into one putative species, thus reducing the risk of overestimating the fungal diversity if only assessed based on ASVs (Kauserud, 2023). For the taxonomic classifications of the OTUs, we considered the plausible identifications with a ≥50% probability threshold (Somervuo et al., 2017). In the resulting data, rarefaction curves at the sample level reached a plateau, indicating a sufficient sequencing depth (Appendix S1: Figure S2).

To evaluate the possibility of cross‐contamination, we included in the sequencing run a total of 162 negative controls (52 negative controls for PCR, 26 negative controls for DNA extraction, and 84 blank samples). The nine target species were not found in any of these 162 negative controls. Furthermore, in those 259 cases in which the nine target species were found in the field samples, they were typically present in a high number of sequences (mean = 2116, min = 4, max = 108,960; in total, five samples in which the species was represented by less than 10 sequences). The absence of the target species in negative controls and their high representation in field samples suggests that cross‐contamination was rare, and hence, our results on colonization success are likely to be robust.

### Statistical analyses

The data consisted of 4662 fungal OTUs identified in 1380 sampling units (i.e., the 460 logs sampled three times). For the statistical analyses, we removed 11 sampling units for which the read count was considered low (<10,000; Appendix S1: Figure S3), resulting in 1369 sampling units. To ask which factors influenced colonization success, we applied probit regression separately for each target species with the R package Hmsc (Tikhonov et al., 2022). For this, we considered the 40 target logs in which inoculation was attempted for each target species as the sampling units (for *F. rosea*, *Perenniporia subacida*, and *Skeletocutis odora*, the total number of sampling units was 39, and for *Physisporinus crocatus*, 38, due to the sampling units discarded based on low sequencing depth) and defined a binary response variable where 1 indicated successful colonization (the target species was found from the log one and/or two years after inoculation) and 0 indicated unsuccessful colonization.

Our main goal was to infer how the resident community influenced the colonization success of each inoculated species, on top of the influence of the abiotic environmental variables. To address this question, we constructed alternative models that included either only abiotic environmental predictors (called henceforth environment‐only model) or additionally different aspects of the resident fungal communities as biotic predictors (called henceforth resident community models) (Table 1). The environment‐only model included log type (categorical variable, levels broken, uprooted, and felled) and decay stage (continuous variable) as environmental abiotic predictors. We controlled for variation in sequencing depth across sampling units directly by modeling it rather than using rarefied values (McMurdie & Holmes, 2014) and included sequencing depth as a predictor (continuous variable, logarithm of the total number of sequences per sample, averaged over the 2 years of sampling after inoculation). Additionally, we included the random effect of site (categorical variable, five levels). We then defined nine alternative resident community models based on community data collected before the inoculations. Biotic predictors of five resident community models were based on the presence–absence data and on abundance data for four of the models (Table 1). For the latter, we translated the community matrix into relative read abundances (RRAs; Deagle et al., 2019) and raised the number to the power of one‐fourth to reduce variation and hence to exclude highly influential datapoints. A summary of the biotic predictors is provided in Appendix S1: Figure S4.

**TABLE 1:** Description of the 10 probit regression models applied to explain the colonization success of the inoculated fungal species.

Data type for predictors		Model	Predictors
…	1	Environment‐only	Log type, decay stage, and sequencing depth (fixed effects) Site (random effect)
Presence‐absence	2	Total species richness	The total no. species in the resident community
	3	Per‐phylum species richness	The no. species in the resident community belonging to *Ascomycota* and *Basidiomycota*
	4	Community composition	The first two latent variables of the model‐based ordination based on the presence–absence version of the resident community matrix
	5	Ten most common species	The occurrences of the 10 most common species in the resident community
	6	Ten most common species (variable selection)	The occurrences of the 10 most common species in the resident community with variable selection applied using slab and spike prior
…	7	DNA amount	The log‐transformed total amount of DNA in the resident community
RRA	8	Community composition	The first two latent variables of the model‐based ordination based on the RRA version of the resident community matrix
	9	Ten most common species	RRA of the 10 most common species in the resident community
	10	Ten most common species (variable selection)	RRA of the 10 most common species in the resident community with variable selection applied using slab and spike prior

*Note*: The environment‐only model includes the abiotic environmental predictors, while the resident community models (2–10) additionally include different aspects of the resident communities as biotic predictors, based on the presence–absence and relative read abundance (RRA) community data.

In the total species richness model, we included the number of all recorded OTUs as a predictor, whereas in the per‐phylum species richness model, we separately included the number of OTUs assigned to phyla *Ascomycota* and *Basidiomycota* to see if the colonization success of inoculated species responded differently to species with distinct evolutionary origins. In fact, species from these two major groups differ systematically in their nutrient uptake strategies (Manici et al., 2024) that may be reflected in distinct influences on the colonizing individuals. For the community composition models, we ran a model‐based ordination with the R package gllvm (Niku et al., 2019) and extracted the loadings for the first two latent variables (LV1 and LV2) to be used as predictors. For the DNA amount model, we calculated the sample DNA amount by first calculating the number of spike reads by subtracting the read count without spikes from the filtered read count and then calculating the proportion between non‐spike and spike reads. We hypothesized that the DNA amount would reflect the overall abundance of the resident fungal community within the log and that the colonization success of inoculated species could decrease with it due to increased competitive interactions and resource limitation. For the 10 most common species models, we focused on the most dominant species within the resident communities and included the presence–absence or RRA of the 10 most common species as separate predictors. The 10 most common species were defined as the 10 species occurring in the highest number of logs among the 40 target logs per species. With the 10 most common species models with variable selection, we additionally applied variable selection with a slab and spike prior approach (see Ovaskainen & Abrego, 2020, pp. 228–241) to determine the best performing combination of predictors.

To estimate and compare the predictive powers of the models, we computed the area under the receiver operating characteristic curve (AUC; Pearce & Ferrier, 2000) based on leave‐one‐out cross‐validation. We also applied variance partitioning to assess the relative contribution of the abiotic and biotic predictors (Tikhonov et al., 2020). Analyses were conducted with R version 4.2.3 (R Core Team, 2023), and the results were visualized with the R package ggplot2 (Wickham, 2016).

## RESULTS

### Overall patterns in resident fungal communities

We recorded 2346 fungal OTUs in the resident communities prior to inoculations. Resident OTU richness per Norway spruce log varied from 2 to 197, with a mean of 52.7 OTUs (Appendix S1: Figure S5). Natural logs held more species‐rich resident communities, with broken logs hosting an average of 59.4 OTUs (ranging from 7 to 147), uprooted logs 56.6 OTUs (ranging from 2 to 197), and felled logs 44.8 OTUs (ranging from 3 to 158). *Ascomycota* and *Basidiomycota* were the most common phyla, representing 57.0% (RRA = 0.63) and 37.9% (RRA = 0.36) of the taxonomically assigned OTUs, respectively.

### Colonization success of the inoculated species

**FIGURE 2:**
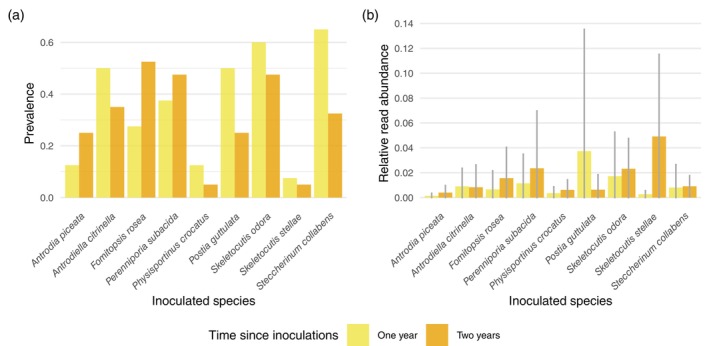
(a) Proportion of target logs where the inoculated target species occurred and (b) their average relative read abundances (RRA) in the target logs one and two years after the inoculations. Each species was inoculated in 40 target logs. Error bars in plot (b) show the SD for RRA.

All inoculated fungal species were detected both one and two years after inoculation in at least some of their target logs (Figure 2). However, prevalence (i.e., proportion of target logs where the species occurred) and RRA depended on the target species. For example, *F. rosea*, *P. subacida*, and *S. odora* occurred in approximately half of their target logs after 2 years, while *P. crocatus* and *Skeletocutis stellae* occurred only in 5% of their target logs (Figure 2a). Most of the inoculated species reached their maximum prevalence 1 year after the inoculation, while only three out of the nine species were detected in more logs 2 years after the inoculations. Nevertheless, seven species showed an increase in average RRA from one to two years after the inoculations (Figure 2b). Overall, colonization success was consistently higher in felled logs than in natural logs (Appendix S1: Figure S6). Target species were rare in the logs where they were not inoculated both before and after the inoculations (Appendix S1: Section S5.1). Thus, their recorded occurrences in the inoculated logs can be attributed to the inoculation treatments.

### Factors explaining the colonization success of inoculated species

Factors affecting colonization success differed among the inoculated species, forming the following three groups of responses. First, for *P. subacida*, *Postia guttulata*, *S. odora*, and *S. stellae*, colonization success was mostly explained by the environmental factors (i.e., log type, decay stage, and the random effect of site) and sequencing depth. All models achieved high predictive power for these species (average AUC based on leave‐one‐out cross‐validation ≥0.75), but the resident community models including different aspects of resident communities as biotic predictors (e.g., total species richness, community composition, or DNA amount) did not perform substantially better than the environment‐only model (Table 2). Second, for *Antrodia piceata*, *F. rosea*, and *Steccherinum collabens*, colonization success was largely explained by the resident communities in addition to the environmental factors. For these three species, not all but several of the resident community models outperformed the environment‐only model (Table 2). Which resident community model showed the best performance varied (Table 2), but generally, the effects of resident communities on the colonization success of these species were negative (Appendix S1: Tables S5 and S6). Third, for *A. citrinella* and *P. crocatus*, models showed poor predictive performance, with all models performing worse than or close to random (average AUC based on leave‐one‐out cross‐validation 0.37 and 0.52; Table 2).

**TABLE 2:** Support for the environment‐only model and different resident community models measured through the area under the receiver operating characteristic curve (AUC) statistic based on leave‐one‐out cross‐validation.

Data type for predictors	Model	Target species								
		*Antrodia piceata* (*n* = 13)	*Antrodiella citrinella* (*n* = 25)	*Fomitopsis rosea* (*n* = 23)	*Perenniporia subacida* (*n* = 21)	*Physisporinus crocatus* (*n* = 6)	*Postia guttulata* (*n* = 24)	*Skeletocutis odora* (*n* = 28)	*Skeletocutis stellae* (*n* = 5)	*Steccherinum collabens* (*n* = 28)
…	Environment‐only	0.66	0.34	0.67	0.75	**0.61**	**0.79**	0.76	0.81	0.73
Presence‐absence	Total species richness	**0.81**	0.26	0.63	0.74	0.53	0.76	0.81	0.78	0.71
	Per‐phylum species richness	0.80	0.39	0.62	0.73	0.48	0.76	**0.82**	0.74	0.72
	Community composition	0.73	0.43	0.66	**0.84**	0.47	0.76	**0.82**	0.85	0.80
	Ten most common species	0.70	0.31	0.74	0.73	0.48	0.78	0.67	0.65	0.88
	Ten most common species (variable selection)	0.61	0.27	**0.81**	0.74	0.51	0.76	0.80	0.78	0.76
…	DNA amount	0.58	0.28	0.66	0.76	0.58	0.73	0.76	**0.92**	0.70
Relative read abundance	Community composition	0.64	**0.60**	0.61	0.75	0.56	0.76	0.75	0.76	0.72
	Ten most common species	0.64	0.40	0.75	0.72	0.38	0.71	0.79	0.63	**0.96**
	Ten most common species (variable selection)	0.60	0.34	0.66	0.76	**0.61**	**0.79**	0.76	0.77	0.82
AUC difference Resident community—Environment only		0.15	0.26	0.14	0.09	0.00	0.00	0.06	0.11	0.23

*Note*: The last row shows the AUC difference between the best supported model among the alternative resident community models and the environment‐only model. The number of sampling units (*n*) in which the inoculations were successful in one and/or two years after the inoculations (*N* = 40) is shown after the name of each inoculated species. Values in boldface show the model with the highest AUC for each target species.

Log type had a statistically supported effect on *P. subacida*, *S. odora*, and *S. stellae*, with the colonization success of the former two being higher in felled logs than in broken logs, and the latter being more successful in broken logs than in uprooted logs (Appendix S1: Table S5). Colonization success of *P. guttulata* increased with decay stage, while *S. odora* showed an opposite trend (Appendix S1: Table S5). Together, log type and decay stage captured 92%, 86%, and 72% of the explained variance in colonization success of *P. subacida*, *S. odora*, and *S. stellae*, respectively, while *P. guttulata* was more evenly affected by all the predictors (log type and decay stage covering 34% of the explained variance, sequencing depth 28%, and site 38%; Appendix S1: Figure S7). None of the resident community models outperformed the environment‐only model for *P. guttulata*, but for *P. subacida*, *S. odora*, and *S. stellae*, some resident community models performed slightly better (at best, AUC increasing by 0.09, 0.06, and 0.11 for the species; Table 2), suggesting that the colonization success of these species was moderately affected by the resident communities.

The total and per‐phylum species richness models were the best supported for *A. piceata* (Table 2). The colonization success of this species decreased with increasing resident total species richness and especially with increasing *Ascomycota* richness (Appendix S1: Table S5). Total and per‐phylum species richness both captured 67% of the explained variance, while the remaining variance was attributed to the abiotic environmental factors in both models (Appendix S1: Figure S7). *A. piceata* obtained high predictive power also for the community composition model based on resident species' occurrences (Table 2). The colonization success of the species was negatively associated with the first latent variable (LV1) summarizing community composition (Appendix S1: Table S5). The two latent variables captured 58% of the explained variance (Appendix S1: Figure S7).

For *F. rosea*, the model including the presence of the 10 most common resident species with variable selection was the best supported (Table 2). The models with the 10 most commonly occurring and abundant species without variable selection also outperformed the environment‐only model (Table 2). The colonization success of *F. rosea* was negatively affected by the presence and RRA of OTU18 (*Fomitopsis pinicola*) and OTU26 (*Ascocoryne cylichnium*) and the RRA of OTU32 (*Collophora* sp.) and OTU40 (*Kuraishia capsulate*) (Appendix S1: Table S6). Depending on the model, the proportion of explained variance attributed to the 10 most common species ranged from 50% to 68% (Appendix S1: Figure S7).

The best supported model for *S. collabens* was the 10 most common species model without variable selection based on RRA (Table 2). Both the model with the 10 most commonly occurring species without variable selection and the model with the 10 most abundant species with variable selection obtained higher predictive power than the environment‐only model (Table 2). The models showed both positive and negative effects of the resident species. Colonization success of *S. collabens* showed positive responses to the RRA of OTU02 (*Collophora* sp.), the presence and RRA of OTU05 (*Cladophialophora* sp.), and OTU15 (*Auricularia* sp.), and negative responses to the presence and RRA of OTU03 (*Exophiala* sp.) and the RRA of OTU18 (*F. pinicola*) and OTU26 (*A. cylichnium*; Appendix S1: Table S6). The 10 most common species captured 11%–71% of the explained variance depending on the model (Appendix S1: Figure S7). Finally, the community composition model based on the presence–absence also outperformed the environment‐only model for *S. collabens* (Table 2). Colonization success of this species was negatively associated with the second latent variable (LV2) (Appendix S1: Table S5). The two latent variables captured 18% of the explained variance (Appendix S1: Figure S7).

## DISCUSSION

While both environmental and biotic filtering are known to be important determinants of community assembly (e.g., Götzenberger et al., 2012; Leibold et al., 2004; Nemergut et al., 2013; Vellend, 2010), experimental quantification of their importance in species‐rich communities has rarely been achieved (but see, e.g., Mighell et al., 2019; Székely et al., 2013). Results from our experiment suggested that overall, both environmental and biotic filters play a significant role in structuring these species‐rich communities. The relative roles of these processes, however, appeared to vary notably among species. Furthermore, biotic filtering seemed to operate differently among the target species.

Our results showed that accounting for resident community composition improved the models' ability to predict the colonization success of some target species, indicating that biotic filtering plays a role in the community assembly of wood‐inhabiting fungi. These patterns could arise from direct interactions between the resident species and the inoculated individuals, or from indirect effects resulting from substrate modification of resident communities, which cannot be disentangled from our experiment. Previous experiments have demonstrated that both pairwise interactions (Boddy, 2000; Heilmann‐Clausen & Boddy, 2005; Hiscox et al., 2018), as well as resource modification by resident communities might affect the colonization success of fungi (Boddy & Hiscox, 2016). Our study further suggested that interactions may take place under more complex settings than pairwise interactions (see also Dickie et al., 2012; Leopold et al., 2017): While the colonization success of some species was strongly affected by certain resident species, others were influenced by the collective presence of several species in the resident community. However, to rigorously assess how these collective effects operate requires direct observations from experiments with differing numbers and combinations of potentially interacting species.

The colonization success of all inoculated species depended on the abiotic environmental conditions and particularly whether the logs were felled, naturally broken, or naturally uprooted. Yet, for some of our target species, log type had a weaker effect than previously proposed, while the resident community composition explained a substantial part of their colonization success. This is partially explained by the fact that log type and community composition are interdependent, the physiochemical properties of the logs influencing fungal community composition (Pasanen et al., 2018; Rajala et al., 2012; Saine, Penttilä, Furneaux, et al., 2024) and vice versa (Fukami et al., 2010; Maynard et al., 2018; Rinne et al., 2016). Here, we disentangled the relative effects of these two axes of variation in the focal logs by comparing the performance of alternative models that included either only predictors describing the physicochemical characteristics of the logs or in addition also biotic predictors describing resident community composition. Following this approach, we showed that the biotic predictors provided additional information that explained the inoculation success of some species, on top of that provided by the abiotic predictors. However, it is possible that other finer‐scale predictors than the ones considered in our study, such as log‐level microclimatic conditions (Pouska et al., 2016), would have explained part of the variation captured by the biotic predictors.

The observed species‐specific responses to abiotic and biotic environments indicate that summarizing community‐wide responses into indices may obscure our capability to understand the mechanisms of fungal community assembly. Results from previous experiments on plant communities have also demonstrated highly variable species‐level responses to different abiotic and biotic factors (Jobe & Gedan, 2021; Klanderud, 2008; Saccone et al., 2017). Accounting for this variation may be especially important for wood‐inhabiting, species‐rich communities, where many different species may show idiosyncratic responses (Abrego et al., 2017) that would be obscured by metrics at the community level. Modeling frameworks allowing for both species‐ and community‐level inference from multispecies data, such as joint species distribution modeling (Norberg et al., 2019; Ovaskainen & Abrego, 2020), facilitate a better understanding of the mechanisms underlying community assembly.

Finally, we note the difference between natural fungal colonization and colonization through fungal inoculation. Fungal colonization of deadwood under natural settings starts from monokaryotic mycelium germinating from the spores, which grows for a period until finding compatible mating types to form the dikaryotic mycelium. In our experiment, the inoculated fungi were already at the dikaryotic stage, which have superior survival rates compared with fungi at the monokaryotic stage (Shirouzu et al., 2014). Likewise, the inoculum size, which was much greater in our experiment than under natural conditions, enhances establishment success (Holmer & Stenlid, 1993). Thus, overall, our inoculations provided a higher chance of successful colonization compared with natural colonization events.

## CONCLUSIONS

By combining DNA‐based community surveys with manipulated colonizations of multiple fungal species in a large‐scale, multi‐year field experiment, we were able to quantify the relative contributions of abiotic and biotic environments to the colonization success of target species in natural settings. Our results indicated that species respond idiosyncratically to biotic and environmental filters, emphasizing the need to account for individual species' responses when predicting community assembly. Additionally, our study demonstrated how manipulative experiments can illuminate the mechanisms underlying community assembly in ways that observational data alone cannot. The roles of other assembly processes, such as stochasticity and dispersal, require further experimental testing. Furthermore, studying the effects of the inoculated species on resident communities would provide a more integrative understanding of priority effects in species‐rich systems.

## CONFLICT OF INTEREST STATEMENT

The authors declare no conflicts of interest.

## Supporting information


Appendix S1.

## Data Availability

Data and code (Saine, Penttilä, Fukami, et al., 2024) are available in Zenodo at https://doi.org/10.5281/zenodo.11108552. The raw sequence data are publicly available in the European Nucleotide Archive (ENA) under project accession number PRJEB65749 at https://www.ebi.ac.uk/ena/browser/view/PRJEB65749.
