# Supplementary material for: Idiosyncratic responses to biotic and environmental filters in wood‐inhabiting fungal communities
Source: Ecology. 2025 Feb 12;106(2):e70013. doi: 10.1002/ecy.70013 (PMC11815356; doi:10.1002/ecy.70013)
Supplement: Supplementary file 1 — Appendix S1. [file ECY-106-e70013-s001.pdf]

Appendix S1.

Sonja Saine, Reijo Penttilä, Tadashi Fukami, Brendan Furneaux, Tuija Hytönen, Otto Miettinen, Norman Monkhouse, Raisa Mäkipää, Jorma Pennanen, Evgeny V. Zakharov, Otso Ovaskainen & Nerea Abrego

Idiosyncratic responses to biotic and environmental filters in wood-inhabiting fungal communities

Ecology

Section S1. Description of the experimental sites and study logs

Table S1. Information on the study sites. All study sites were located in southern and central Finland. Sites included state owned forests managed by Metsähallitus (State Forests, Finland), set-aside forests owned by forestry company UPM, and a set-aside forest owned by the city of Helsinki. As explained in the main document, size of the experimental areas varied from 2 to 5 hectares subject to the availability of spruce deadwood filling the criteria for study logs. Experimental area size was calculated as the area (*ha*) covered by the study logs with a 20-meter buffer surrounding each log. Mean stand age (*y*) was obtained by calculating average stand age for an area that covered all study logs with a 50-meter buffer. Data on stand age from Natural Resources Institute Finland (2019).

Site	Municipality	Bioclimatic zone	Site type	Site owner	Experimental area size (*ha*)	Mean stand age (*y*)
Kesijärvi	Janakkala	southern boreal	set-aside forest	UPM	2	58
Lapinjärvi	Lapinjärvi	southern boreal	set-aside forest	Metsähallitus	4	64
Luukki	Espoo	southern boreal	set-aside forest	City of Helsinki	5	89
Seitseminen	Ylöjärvi	middle boreal	national park	Metsähallitus	4	83
Sääjärvi	Janakkala	southern boreal	set-aside forest	UPM	5	72

Table S2. Characteristics of the study logs at each site. We selected both natural and felled logs with a diameter ≥ 20 cm at breast height (1.3 m from the base; DBH). In site Lapinjärvi where large logs were scarcer, we also chose two natural logs with 18 and 19 cm DBH, and for felled logs, one spruce with 18 cm and five with 19 cm DBH. Since felled logs were all recently felled in decay stage 1, we only selected natural logs in decay stages 1 and 2 (on a scale from 1 to 5 where 1 is recently dead, hard wood, and 2 is slightly decayed wood where the knife penetrates 1–2 cm; Renvall 1995). Altogether, each site hosted 55 natural logs and 37 felled logs. Measured log characteristics were log type (broken, uprooted or felled), DBH, decay stage in years 2019 and 2021, the proportion of inoculation area (from the log base up to 11 meters) touching the ground and covered by bark, and the proportion of open canopy above the log. Table shows the proportion of logs in decay stages 1 and 2 (DS1/DS2 %) for decay stages in 2019 and 2021 and for other characteristics, the first quantile, median, and the third quantile (Q1 – median – Q3).

Site	Log type	Mortality factor	n	Decay stage 2019	Decay stage 2021	DBH (cm)	Ground contact (%)	Bark cover (%)	Canopy openness (%)
Kesijärvi	natural	broken	1	100/0%	100/0%	24.0	0	100	49.4
	natural	uprooted	54	56/44%	44/56%	23.0–27.0–29.0	0–10–20	90–90–100	31.2–39.8–53.1
	felled	-	37	100/0%	100/0%	27.0–29.0–32.0	10–20–40	90–100–100	20.8–23.6–30.2
Lapinjärvi	natural	broken	34	85/15%	79/21%	22.0–24.0–28.0	0–10–10	10–35–80	25.0–35.2–45.9
	natural	uprooted	21	86/14%	57/43%	27.0–29.0–36.0	0–10–10	70–80–90	24.5–29.4–38.7
	felled	-	37	100/0%	100/0%	20.0–24.0–26.0	0–10–10	40–80–90	23.5–28.5–31.9
Luukki	natural	broken	30	60/40%	50/50%	25.2–28.0–32.0	0–10–20	20–30–50	20.0–22.5–29.9
	natural	uprooted	25	36/64%	20/80%	32.0–36.0–39.0	0–0–10	10–20–40	20.6–22.6–28.3
	felled	-	37	100/0%	100/0%	27.0–30.0–33.0	0–10–30	30–40–70	19.5–22.6–24.6
Seitseminen	natural	broken	49	71/29%	63/37%	27.0–30.0–33.0	0–10–30	40–70–90	26.0–30.1–35.6
	natural	uprooted	6	67/33%	67/33%	29.2–31.5–33.8	0–5–25	75–90–97.5	29.7–35.2–36.3
	felled	-	37	100/0%	100/0%	25.0–28.0–31.0	10–30–40	90–100–100	23.8–26.4–29.8
Sääjärvi	natural	broken	23	78/22%	70/30%	22.0–24.0–26.0	5–10–20	5–20–50	17.2–32.8–40.4
	natural	uprooted	32	50/50%	47/53%	22.8–26.5–30.0	0–10–20	27.5–55–90	23.0–28.3–36.8
	felled	-	37	100/0%	100/0%	26.0–28.0–31.0	10–20–40	70–90–100	21.3–26.1–34.8

Section S2. Information on target species, fruit-body collections, strain cultivations, and inoculations

Section S2.1. Target species' distribution and conservation status

Target species belonged to the families *Dacryobolaceae, Fomitopsidaceae, Incrustoporiaceae, Meripilaceae, Polyporaceae* and *Steccherinaceae*, all within the order *Polyporales* (Justo et al. 2017). Known distributions of the species cover the studied areas (Table S3) but they have declined strongly especially in southern Finland (Kotiranta et al. 2019). In the latest red-list assessment of Finnish species in 2019 (Hyvärinen et al. 2019), one target species was listed as endangered and two as vulnerable, and the rest were classified as near threatened (n = 5) and least concern (n = 1; but NT in the previous Finnish red-list, see Kotiranta et al. 2010) (Kotiranta et al. 2019; Table S3). For most target species (all except *Postia guttulata*), lack of deadwood and old-growth forests were listed as the main causes of threat. All target species use Norway spruce as their primary host (Table S3).

Table S3. Information on the inoculated target species. Information on rot type, host tree species and distribution follows Niemelä (2016), and red-list statuses the 2019 Red List of Finnish Species (Hyvärinen et al. 2019; Kotiranta et al. 2019). Threatened species are marked with an asterisk.

Species	Rot type	Host tree species in Finland	Distribution in Finland	Red-list status
Antrodia piceata	brown	spruce	from southern Finland to Sodankylä's Lapland	vulnerable*
Antrodiella citrinella	white	spruce, rarely birch, aspen, alder	the whole of Finland	near threatened
Fomitopsis rosea	brown	spruce, rarely pine and kelo aspen	the whole of Finland	near threatened
Perenniporia subacida	white	spruce, rarely pine, birch, and aspen	the whole of Finland	near threatened
Physisporinus crocatus	white	birch, alder, coniferous trees	southern and south-eastern Finland, eastern species	endangered*
Postia guttulata	brown	spruce, pine	up to the southern part of Northern boreal zone	least concern
Skeletocutis odora	white	spruce, aspen, rarely pine	the whole of Finland	near threatened
Skeletocutis stellae	white	spruce, pine	the whole of Finland	vulnerable*
Steccherinum collabens	white	spruce, rarely aspen	the whole of Finland, more abundant in the north	near threatened

Section S2.2. Fruit-body collections, strain cultivations, Sanger-sequencing, and inoculations

Strains for the experiment were obtained by sampling fungal populations in the field, as well as from the University of Helsinki fungal culture collection (Table S4). In August–November 2018, the field sampling for the experiment was conducted in 18 forest sites belonging to 14 municipalities in southern and central Finland (Table S4; Fig. S1). All visited forest sites were protected.

With pileate and thick resupinate species, we sampled a piece of fruit-body, cut a small piece from the inside of fruit-body, transferred it to a malt extract agar plate (2%) and sealed the plate. With thin resupinate species, we sampled a piece of fruit-body and taped it on the lid of a malt extract agar plate, closed and sealed the plate, and kept it closed overnight to allow agar gel to catch the spore deposition. In both cases, the source organisms were not destroyed by sampling (Nordén et al. 2020).

To confirm the species identifications, every strain was Sanger-sequenced. After cultivation on malt extract agar plates, the strains were transferred to MOS agar plates for DNA extraction. Isolates were sampled by scraping the fungus into a garnet bead tube, homogenized using FastPrep homogenizer, and stored at -80 °C. The samples were extracted using the E.Z.N.A.® Forensic DNA Isolation Kit (WVR D3591-02). PCR amplification of the ITS region was performed using primers ITS1F (F) (CTTGGTCATTTAGAGGAAGTAA; Gardes and Bruns 1993) and ITS4 (R) (TCCTCCGCTTATTGATATGC; White et al. 1990). For the ITS PCR, 1 µl of 1:100 diluted DNA extract was added to a mixture consisting 12.5 µl Dreamtaq Green PCR Master Mix (2X), 0.125 µl of each primer (25 µM) and 11.25 µl of PCR grade water. The PCR included the following cycling conditions: initial denaturation at 95 °C for 3 minutes, followed by 35 cycles of 30 seconds denaturation at 95 °C, 30 seconds at 55 °C, and 1 minute extension at 72 °C, followed by final extension at 72 °C for 10 minutes. After PCR, the amplicons were run on a 1% agarose gel and purified. Sequencing was performed at Macrogen using primer ITS1F. After sequencing, the sequences were trimmed and filtered using Geneious Prime software. Taxonomic classification was performed using Unite and Nucleotide programs.

During the field experiment, inoculations were conducted using 2–5 strains per species depending on the total number of strains available for target species (Table S4). During the inoculation phase, the inoculation dowels for one out of five original strains of *Skeletocutis odora* and one out of three original strains of *Antrodiella citronella* became visibly contaminated and additional strains were obtained as replacements. Later, one out of three strains of *Skeletocutis stellae*, one out of three strains of *Antrodiella citrinella*, and one out of two strains of *Physisporinus crocatus* became contaminated and were not used for the remaining inoculations.

Table S4. Target species and their strains used for the inoculations. Strains were obtained either by sampling fungal population in the field (*origin = field*) or from the University of Helsinki fungal culture collections (*origin = col.*). Sampling location shows the site, municipality, and country if available. Samplings were conducted in Finland (FIN) and Estonia (EST). Growth time is the time it took for mycelia to fill an agar plate (with a diameter of 90 mm) in the lab in days.

**Species**	**Strain ID**	**Origin**	**Sampling location**	**Sampling date**	**Growth time**
Antrodia piceata	FBCC2616	col.	Hyytiälä, Ruovesi FIN	2008-09-15	-
	FBCC2617	col.	Hyytiälä, Ruovesi, FIN	2008-09-15	-
	JPC150	field	Koivusuo nature reserve, Ilomantsi, FIN	2018-09-27	16
Antrodiella citrinella	JPC108	field	Vesijako nature reserve, Padasjoki, FIN	2018-09-21	15
	JPC199	field	Musturi, Ruovesi, FIN	2018-10-18	51
	JPC207	field	Nuijakorpi primeval forest, Ruovesi, FIN	2018-10-18	63
	OMC1724	col.	Paljakka nature reserve, Hyrynsalmi, FIN	2018-06-27	16
Fomitopsis rosea	JPC64	field	Ulvinsalo nature reserve, Kuhmo, FIN	2018-09-05	19
	JPC143	field	Koivusuo nature reserve, Ilomantsi, FIN	2018-08-26	22
	JPC162	field	Patvinsuo national park, Lieksa, FIN	2018-09-29	24
	JPC196	field	Sinivuori nature reserve, Orivesi, FIN	2018-10-12	25
	JPC228	field	Metsäkulma conservation area, Mäntsälä, FIN	2018-11-05	20
Perenniporia subacida	FBCC523	col.	-	-	-
	JPC71	field	Haukiniemi, Savonranta, FIN	2018-09-11	11
	JPC105	field	Vesijako nature reserve, Padasjoki, FIN	2018-09-21	22
	JPC201	field	Nuijakorpi primeval forest, Ruovesi, FIN	2018-10-18	45
	JPC236	field	Tammisto conservation area, Vantaa, FIN	2018-11-06	21
Physisporinus crocatus	JPC116	field	Vesijako nature reserve, Padasjoki, FIN	2018-09-21	18
	OMC1703	col.	Lemmjõe, Põhja-Sakala vald, EST	2018-09-17	22
Postia guttulata	JPC73	field	Raatelamminsalo, Savonranta, FIN	2018-09-12	47
	JPC96	field	Kotinen, Hämeenlinna, FIN	2018-09-18	74
	JPC120	field	Vesijako nature reserve, Padasjoki, FIN	2018-09-21	35
	JPC144	field	Koivusuo nature reserve, Ilomantsi, FIN	2018-09-27	-
Skeletocutis odora	JPC8	field	Heinäpuro, Sotkamo, FIN	2018-08-23	57
	JPC32	field	Paljakka, Puolanka, FIN	2018-08-26	29
	JPC63	field	Ulvinsalo nature reserve, Kuhmo, FIN	2018-09-05	17
	JPC65	field	Haukiniemi, Savonranta, FIN	2018-09-11	57
	JPC95	field	Kotinen, Hämeenlinna, FIN	2018-09-18	21
	JPC97	field	Kotinen, Hämeenlinna, FIN	2018-09-19	31
Skeletocutis stellae	FBCC2222	col.	Paljakka, Hyrynsalmi, FIN	2010-09-27	-
	JPC185	field	Multiharju, Ikaalinen, FIN	2018-10-09	61
	OM21578	col.	Paljakka, Hyrynsalmi, FIN	2018-06-27	-
Steccherinum collabens	JPC104	field	Metsäksensoidinmaa, Hämeenlinna, FIN	2018-09-20	22
	JPC109	field	Vesijako nature reserve, Padasjoki, FIN	2018-09-21	28
	JPC125	field	Vesijako nature reserve, Padasjoki, FIN	2018-09-21	22
	JPC197	field	Kalvola, Hämeenlinna, FIN	2018-10-15	17
	JPC233	field	Herukkapuro conservation area, Vantaa, FIN	2018-11-06	25

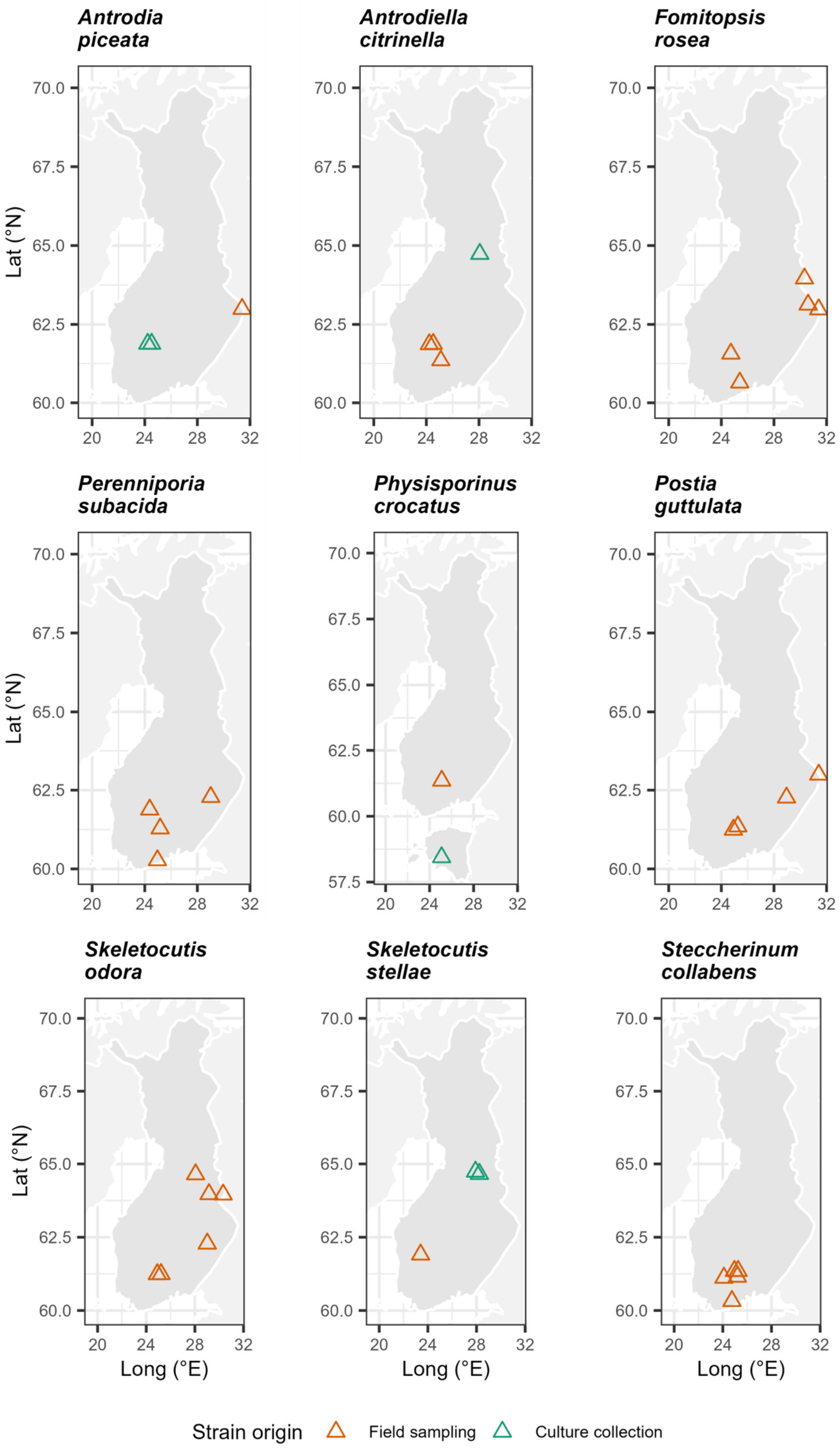

Figure S1. Maps showing the locations where the strains for each target species were collected in Finland and Estonia. Orange triangles show sampling locations for strains that were obtained for the experiment by sampling fungal populations in the field. Green triangles show sampling locations for strains that were obtained from the University of Helsinki fungal culture collections (not shown for one strain of *P. subacida* for which sampling locations was unavailable).

Section S3. Methods for sample pre-processing, DNA extraction, sequencing, and bioinformatic analyses

Section S3.1. Sample pre-processing

Samples were pre-processed at the University of Helsinki, Finland. Sawdust samples collected in 2019 were stored in freezer at -20°C after collection in the field. These samples were collected separately for each inoculation point within a log and thus, we pooled the inoculation point-specific samples to obtain log-level samples. This was done by taking a same-sized subset of a frozen sample with sterile tweezers and combining them in a 50 ml tube, resulting in a pooled sample of ca. 20 ml. The samples were then returned to the freezer. Samples collected in 2020 and 2021 were pooled at the log level already in the field by collecting sawdust from all ten inoculation points in one ziplock bag for each log. After returning from the field, we transferred a 20 ml subset of each sawdust sample into a 50 ml tube with a clean disposable teaspoon. The sample tubes were stored in freezers (-20°C). When sample collection for a given year was completed, all samples were freeze-dried. We took the sample tubes from the freezer, sealed them with parafilm, pricked holes in parafilm with a needle and placed them in a freeze dryer (Christ GAMMA 2-16 LSC) for 62–93 h (at 0.57 mbar vacuum and 15°C self-temperature). As a final pre-processing step, the dried sawdust samples were pulverized. The samples and a mix of sterile metal beads (6 x 4 mm and 2 x 10 mm in diameter) were placed in metallic grinding jars (25 or 50 ml) using sterile tweezers, and ground for five minutes at a 30 s^{-1} frequency in a homogenizer (Mixer Mill MM 400, Retsch). This was repeated until the samples were fully homogenized. A subset of a homogenized sample was placed in a 5 ml tube (for 2019 samples, a 2–4 ml subset, and for 2020 and 2021 samples, a 1 ml subset). The grinding jars were cleaned after each sample by rinsing with water, drying with air blower, spraying with a DNA/RNA decontamination solution (PDS-250, Biosan SIA) and wiping with clean paper towels. Metal beads and tweezers were washed and then cleaned of any remaining DNA by dry heating for four hours at 200°C.

Section S3.2. DNA extraction and sequencing

Sample lysis, DNA extraction, and PCR amplification were done at the Canadian Centre for DNA Barcoding (CCDB), and next generation sequencing of indexed amplicon libraries was completed at the Advanced Analysis Centre at the University of Guelph, Canada. The three annual set of samples were registered under accessions CCDB-22-0650, CCDB-22-0651, and CCDB-22-0652 at the CCDB. Each set of samples was submitted with a plate record associating each sample with a well in a 96-well sample array. The 5 ml tubes were arrayed in tube racks to match the order in a corresponding plate record and their external surfaces were sterilized with 70% ethanol. We spun down the samples for 2 minutes at 5000g in preparation for lysis. To each sample, we added 2–4 ml of insect lysis buffer (ILB) depending on the amount of sawdust in the tube together with 1% polyvinylpyrrolidone (PVP) and 25 uL of Proteinase K (20 mg/ml) per 1 mL of buffer. Then, the samples were centrifuged for 2 minutes at 2000g, placed in a rack on a shaker at 100 RPM and incubated at 56°C for 2 hours followed by a 2-hour incubation at 65°C.

DNA extraction follow the procedure described in Ovaskainen et al. (2020) applied for the Global Spore Sampling Project (GSSP). Tubes with the lysates were centrifuged for 5 minutes at 2000g. A volume of 100 ul was subsampled into a 1 ml deep-well plate (Eppendorf Cat No) with a single channel pipette and mixed with 200 ul of 5M GuSCN Plant Binding buffer. The 300 ul mixture was transferred onto a 96-well 1 um Glass Fiber (GF) plate (PALL) to bind DNA with the membrane and centrifuged for 5 min at 5000g. For the first and second DNA wash, samples were centrifuged at 5000g for 2 minutes with 300 ul of 5M GuSCN buffer followed by 300 ul of Plant Protein Wash buffer. We repeated the final wash twice with 600 ul of Wash buffer and centrifuging at 5000g for 5 minutes. Then, the GF plate was incubated at 56°C for 30 minutes. DNA was eluted from dried membrane with 70 ul of 10mM TrisHCL pH 8.0 and centrifugation at 5000g for 5 minutes.

For the PCRs, we employed a 96-well format with 10.5 ul of standard CCDB Platinum Taq Master Mix. Extracted DNA was used as template for amplification by transferring 2 ul of sample to a template-free reaction-ready premade PCR 1 plate with primers ITS3-misN6 and ITS4-misN6 from Ovaskainen et al. (2020). As a

spike-in, a cocktail of nine synthetic controls was used in every sample (at 0.001 ng/ul). Wells H1 and H12 served as negative controls and were thus left without DNA template. For PCR 1, the following cycling conditions were applied: initial denaturation at 94°C for 2 minutes, followed by 40 cycles of 40 seconds denaturation at 94°C, 1 minute annealing at 51°C, and 1 minute extension at 72°C, followed by final extension at 72°C for 5 minutes. PCR 2 (indexing) involved the same mastermix but contained fusion primers with standard i5 and i7 Illumina indices (N701, N702, N703, N704, N705, N706, N707, N710 and S502, S503, S505, S506, S507, S508, S510, S511, S513, S515, S516, S517). PCR 1 products were diluted 1:1 and 2 ul of the diluted product was used as template in PCR 2. PCR 2 applied the following cycling conditions: initial denaturation at 94°C for 2 minutes, followed by 20 cycles of 40 seconds denaturation at 94°C, 1 minute annealing at 60°C, and 1 minute extension at 72°C, followed by final extension at 72°C for 5 minutes. Transfers of DNA template and indexed primers were done on a Biomek FXP robot.

After PCR, we visualized the amplicons on precast agarose gels using a bufferless E-gel system (Invitrogen). Before sequencing, the amplicons from each well were pooled without normalization, purified using AMPure beads, quantified on a Qubit 2.0 fluorometer, and checked for size on Agilent Bioanalyzer with high sensitivity kit. Sequencing was performed on Illumina MiSeq with PE2x300 following standard manufacturer’s protocol.

Section S3.3. Bioinformatic analyses

For the bioinformatic analyses, we used a development version of the OptimOTU pipeline (available at https://doi.org/10.5281/zenodo.11108552, deadwood_priority_effects.zip) that was implemented in R version 4.2.2 (R Core Team 2022) using the targets workflow management package, version 0.14.2 (Landau 2021). Trimming and filtering was conducted with Cutadapt version 4.2 (Martin 2011). The paired-end fastq files were truncated at the first base with a quality score ≤ 2 at both ends of R1, and at the first base with a quality score ≤ 2 for the 3’ end of R1, and quality score ≤ 10 for the 5’ end of R2. Then, we trimmed both reads by removing multiplexing indices at both ends, with the presence at the 3’ end coded as optional. We then removed both reads if either of them contained “N” bases or had a length < 100bp. Next, an additional filtering round was performed with DADA2 version 1.26 (Callahan et al. 2016). We removed read pairs with R1 having more than 3 expected errors or R2 having more than 5 expected errors and reads mapping to the PhiX genome. Then to dereplicate, denoise, merge, and chimera-check the reads separately for each run, we followed the standard DADA2 ITS pipeline (Callahan 2020). Reads corresponding to the SynMock spike sequences were identified using the -usearch_global command in VSEARCH version 2.22.1 (Rognes et al. 2016) with pairwise identity threshold 0.9. We counted the number of spike sequences for each sample, and used this information to estimate DNA amount in each sample by calculating the proportion between non-spike and spike reads.

To taxonomically identify the remaining ASVs with a 50% probability threshold, we applied Protax-Fungi (Abarenkov et al. 2018). We clustered the ASVs using taxonomically informed pseudo-single-linkage clustering in three phases. In the first phase, we joined all ASVs identified to the same taxon at the current rank to form reference cluster cores. In the second phase, we used the -usearch_global command in VSEARCH to match and join unidentified sequences to the cluster cores. To generate approximately single linkage clusters, we repeated this phase on the growing cluster until no new matches were found. However, we kept taxonomically distinct ASVs in separate clusters by never merging cluster cores. In the third phase, we single-linkage clustered the remaining ASVs by using OptimOTU 0.6.4 (available at https://doi.org/10.5281/zenodo.11108552, optimotu_v0.6.4.zip) to process a sparse distance matrix based on global alignments. We calculated the matrix using the calc_distmx command in USEARCH version 11.0.667 (Edgar 2010). Finally, to determine optimal clustering threshold at each rank, we used the hierarchical optimization technique developed by Dnabarcoder (Vu et al. 2020) except using the same USEARCH + OptimOTU single-linkage clustering. As references for the threshold optimization, we used taxonomically identified fungal sequences from the GSSP (Ovaskainen et al. 2020). These sequences use the same primers and hence cover the same amplicon region as our data. Since Protax-Fungi does not identify non-fungi, we used the -usearch_global command in VSEARCH to find the closest match to each ASV sequence in the Unite sh_matching pipeline Sanger references dataset (Abarenkov 2022), with minimum match identity 0.8, to identify ASVs which belonged to non-fungal groups. We then

removed phylum-level cluster containing more known non-fungi than known fungi. We assigned unique placeholder names to taxonomically unidentified clusters at each rank after clustering. Placeholder names followed the form of “pseudo{rank}_NNNNN”, e.g., “pseudogenus_00132”.

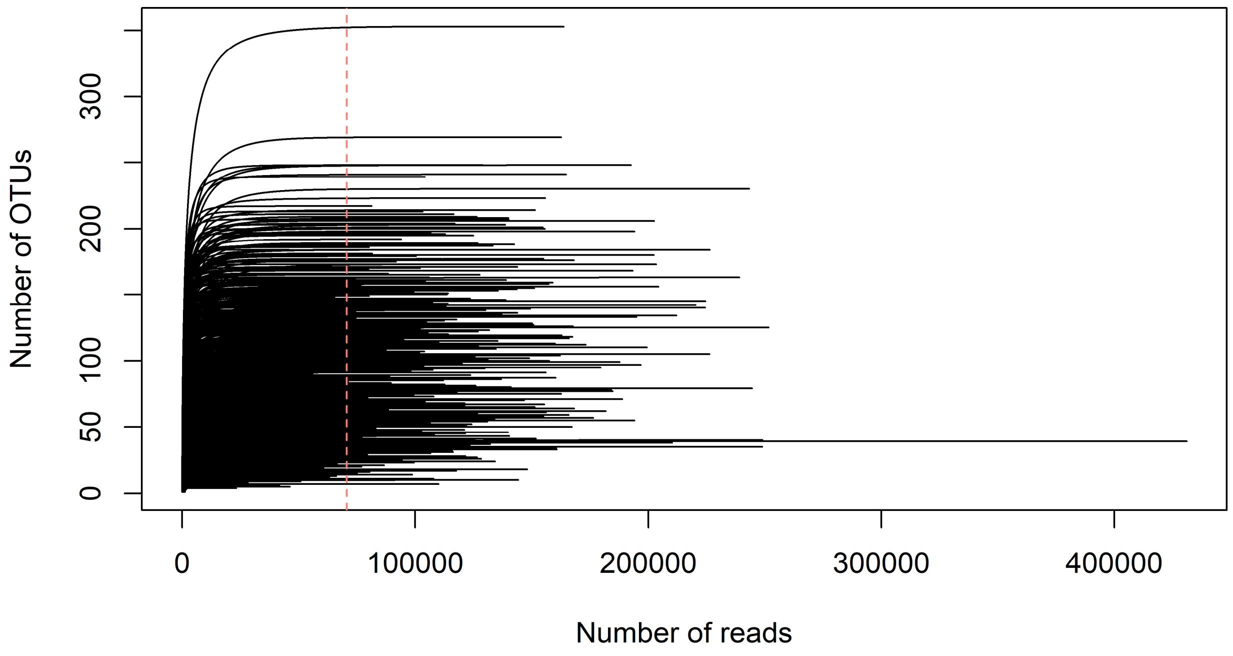

Figure S2. Rarefaction curves for each sampling unit (n = 1375, sampling units with no fungal reads excluded) showing the relationship between the number of reads (i.e., sequencing depth) and the rarefied number of OTUs. Red dashed line shows the mean read count.

Section S4. Statistical analyses

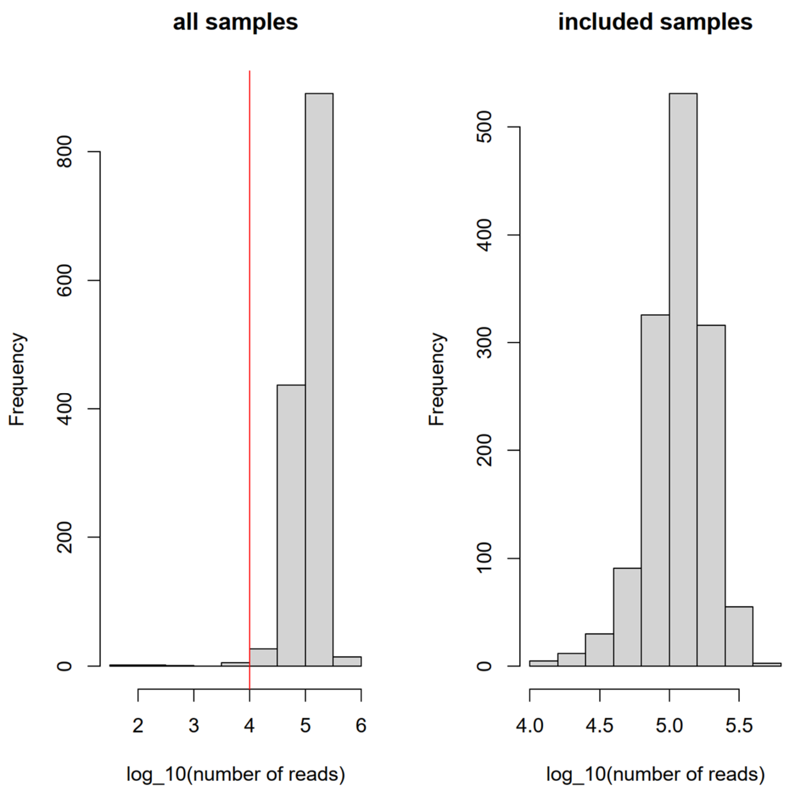

Figure S3. Distribution of total number of reads among all samples (left-hand panel) and those samples that were included in the statistical analyses (right-hand panel). The vertical red line in the left-hand panel shows the cut-off set to 10,000 sequences.

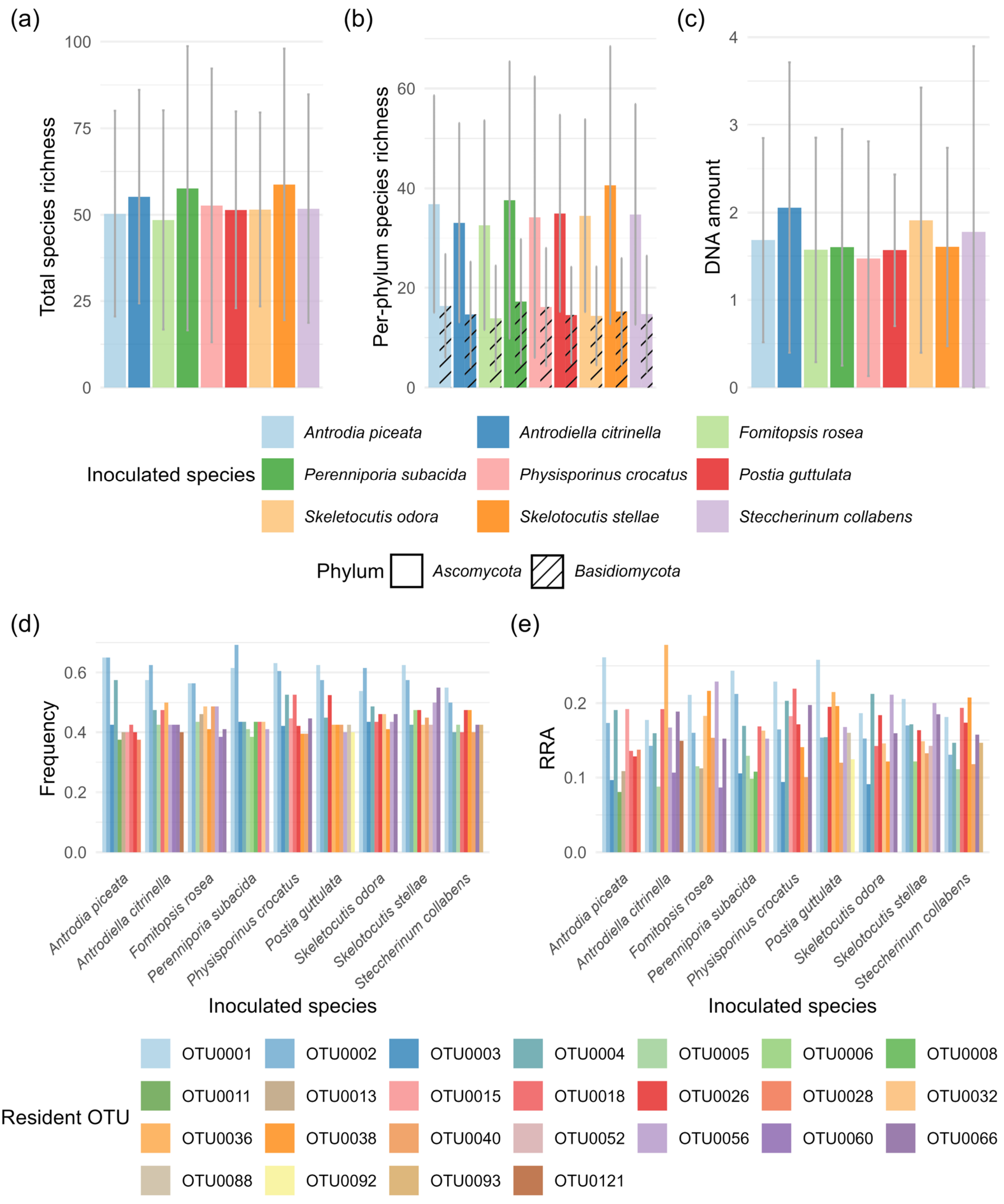

Figure S4. Summary of different aspects of the resident fungal communities included in the resident community models (Table 1) separately for each inoculated species: (a) total species richness, (b) per-phylum species richness (species richness separately for OTUs assigned to phylum *Ascomycota* and *Basidiomycota*), (c) DNA amount (not log-transformed), and (d) frequency and (e) relative read abundance (RRA) of the ten most common OTUs in the resident communities. All variables are averages across the logs for each inoculated species, with error bars showing the standard deviations in plots (a), (b), and (c). Plots (a), (b), and (d) are based on the presence-absence data, while plot (e) is based on the relative read abundance data.

Section S5. Supplementary results

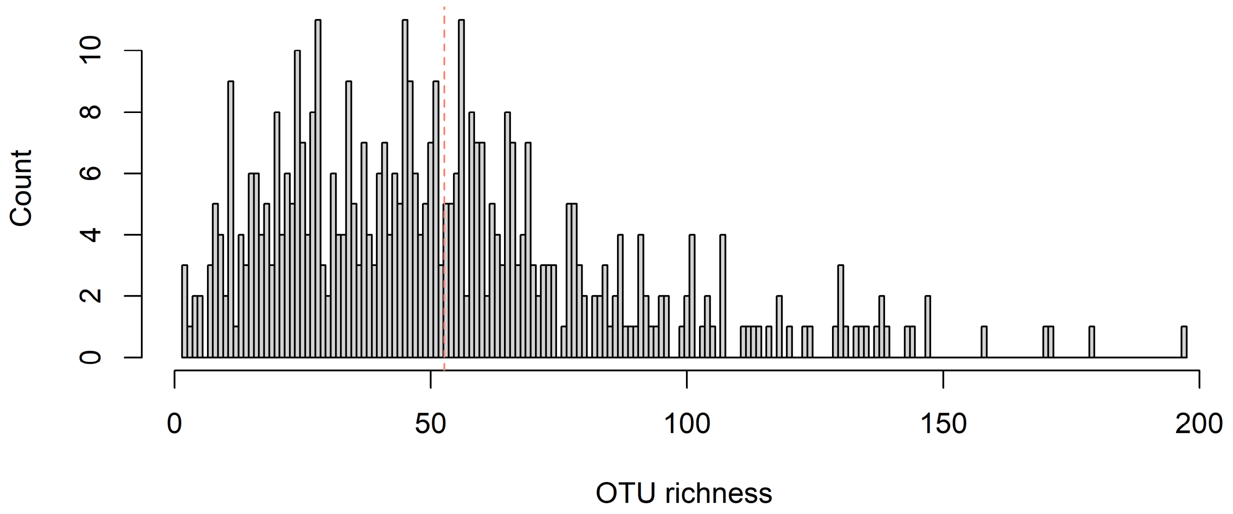

Figure S5. Histogram showing the number of resident OTUs per sampling unit (n = 457) in logs before the inoculations. Red dashed line shows the mean OTU richness.

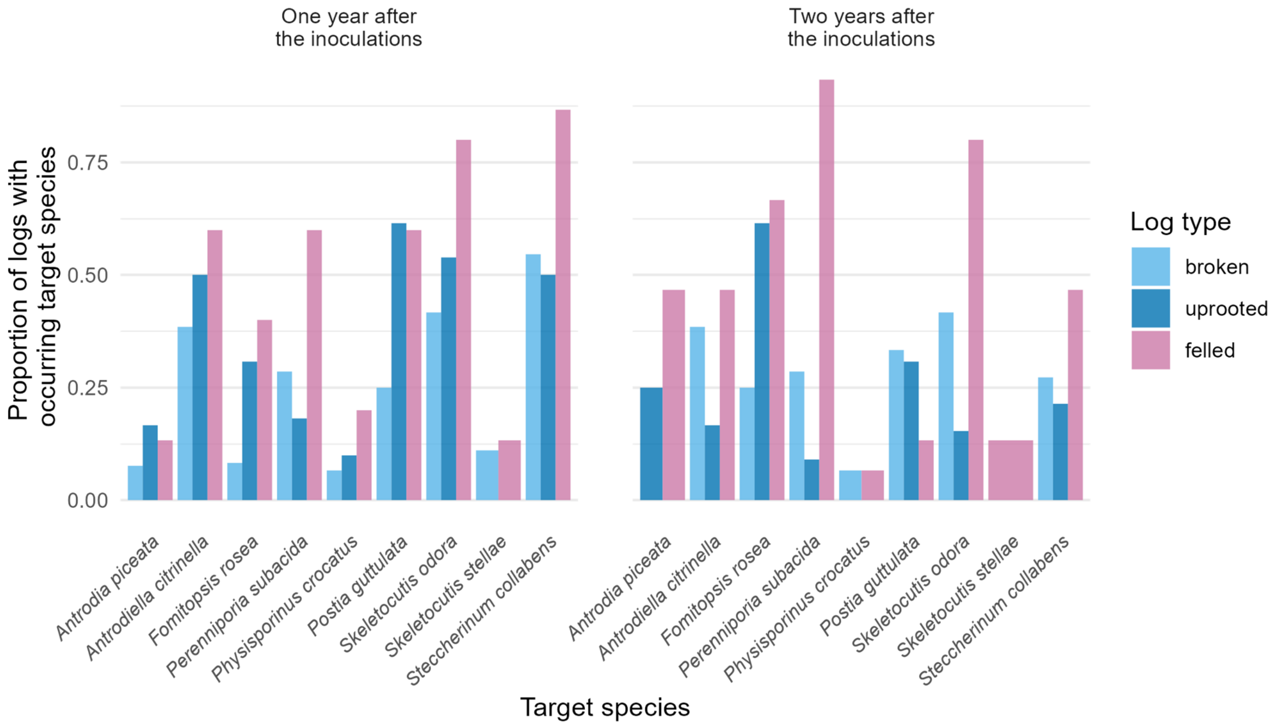

Figure S6. Proportion of logs where the inoculated target species occurred one and two years after the inoculation separately for natural and felled logs. Each species was inoculated in 25 natural logs (including both broken and uprooted logs) and 15 felled logs.

Section S5.1. Natural occurrences of the inoculated target species

Both before and after the inoculations, occurrences of inoculated target species in logs where they were not inoculated in were rare. *Skeletocutis odora* occurred in the resident community of one log before the inoculations of that species. However, the sampling unit was not removed from the data as *Skeletocutis odora* did not occur in that log during later years. Regarding the occurrence of target species in logs where they were not inoculated in, *Fomitopsis rosea* and *Steccherinum collabens* occurred in 3.0% and 2.0% of control logs, and 1.4% and 0.6% of logs where other target species had been inoculated, respectively (across all years). In the same way, *Postia guttulata* was recorded in 0.8% of the logs inoculated with other target species, and *Perenniporia subacida* and *Skeletocutis odora* both occurred in 0.3% of logs inoculated with other target species.

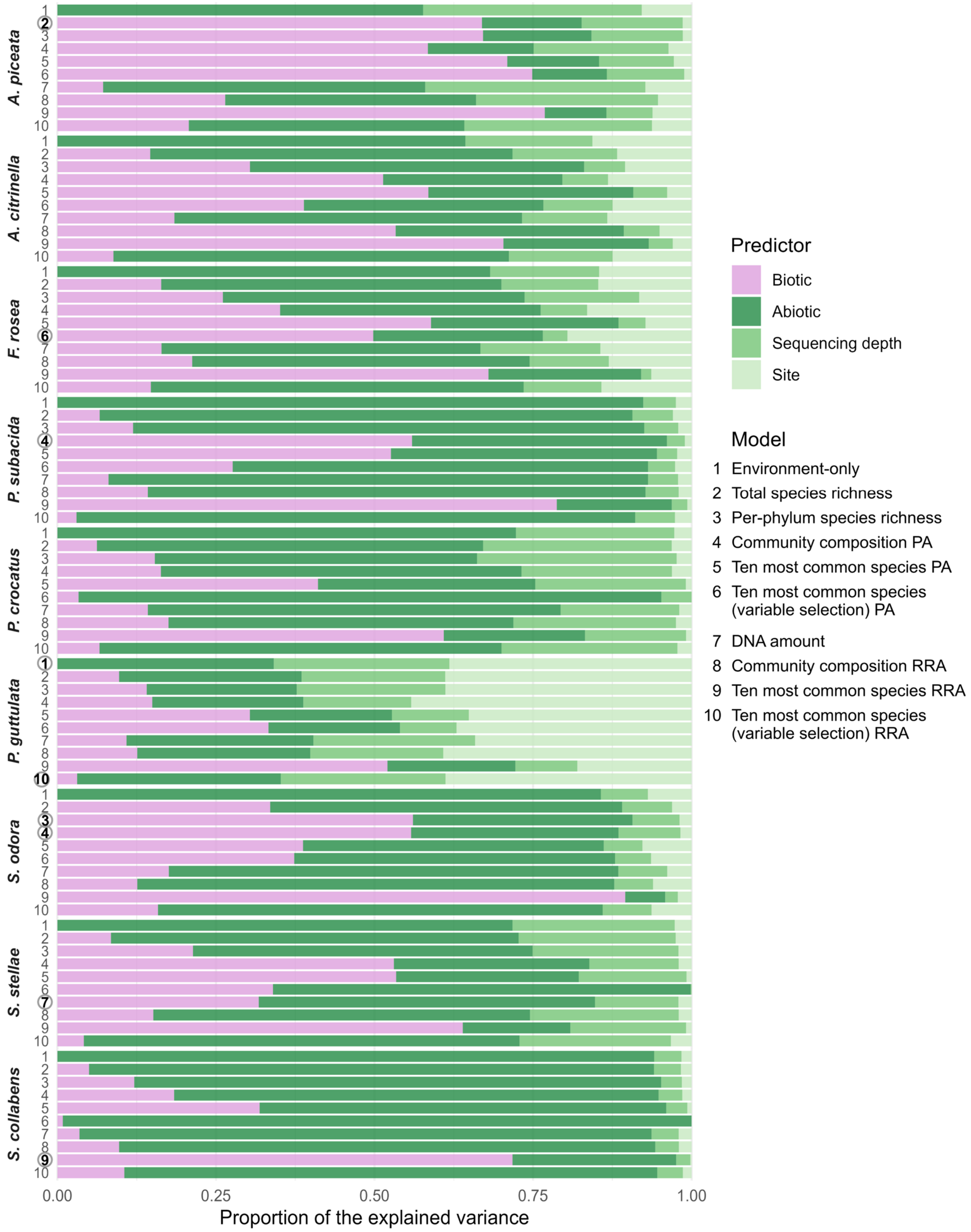

Figure S7. Results for the variance partitioning showing the proportion of explained variance attributed to model predictors in alternative models for each inoculated species. Environment-only model (1) included only the environmental and technical predictors (log type, decay stage, sequencing depth, and site), while models 2–10 additionally included different aspects of a resident fungal community as a biotic predictor. Predictor *Abiotic* shows the total explained variance attributed to log type and decay stage. If the resident fungal community was represented by more than one biotic predictor, the bar for predictor *Biotic* shows the sum of these proportions. Models 2–6 are based on presence-absence (PA) and models 7–10 on relative read abundance (RRA) data on the resident communities. The number for the best supported model(s) based on AUC and leave-one-out cross-validation is bolded and circled (except for *A. citrinella* and *P. crocatus* with the average AUC 0.37 and 0.52).

Table S5. Estimated regression parameters (Beta) and their posterior probabilities (Pr(Beta>0)) from the environment-only model and alternative resident community models, separately for each inoculated species. Beta parameters describe how the target species respond to each predictor. Environment-only model included fixed factors log type (levels felled and uprooted, with broken log type included as an intercept), decay stage, and sequencing depth as predictors. In addition to these environmental abiotic predictors, each resident community model included biotic predictor(s) describing alternative aspects of resident communities described in Table 1 of the main document: total species richness, per-phylum species richness (results separately for phyla *Ascomycota* and *Basidiomycota*), community composition based on presence absence (PA) and relative read abundance (RRA) (results separately for the first (LV1) and second (LV2) latent variables), and DNA amount. Positive (negative) responses to predictors with at least 0.95 (0.05 or less) posterior probability are indicated with darker red (blue), and responses with at least 0.90 (0.10 or less) posterior probability are indicated with lighter red (blue).

		**Inoculated species**								
**Model and predictors**		*Antrodia piceata*	*Antrodiella citrinella*	*Fomitopsis rosea*	*Perenniporia subacida*	*Physisporinus crocatus*	*Postia guttulata*	*Skeletocutis odora*	*Skeletocutis stellae*	*Steccherinum collabens*
Environment-only										
Log type: felled	Beta	**1.059**	0.393	0.704	**1.489**	-0.197	0.382	**1.667**	0.468	0.754
	Pr	**0.986**	0.792	0.922	**1.000**	0.354	0.763	**0.993**	0.777	0.905
Log type: uprooted	Beta	0.391	-0.016	0.749	-0.380	-0.030	-0.241	0.141	-1.370	-0.047
	Pr	0.744	0.499	0.907	0.254	0.480	0.369	0.617	0.058	0.460
Decay stage	Beta	-0.449	-0.533	**-1.141**	-0.680	**-2.365**	**1.372**	**-1.096**	-1.123	**-4.099**
	Pr	0.306	0.174	**0.043**	0.138	**0.016**	**0.988**	**0.030**	0.242	**0.000**
Sequencing depth	Beta	**1.158**	-0.275	0.665	-0.135	1.143	**1.610**	-0.447	**1.649**	0.401
	Pr	**0.952**	0.322	0.860	0.405	0.944	**0.991**	0.254	**0.978**	0.717
Total species richness										
Species richness	Beta	**-0.041**	0.003	-0.011	0.004	-0.003	0.006	**-0.027**	0.003	-0.003
	Pr	**0**	0.656	0.059	0.725	0.382	0.760	**0.006**	0.622	0.365
Log type: felled	Beta	**0.954**	0.398	0.473	**1.619**	-0.249	0.457	**1.786**	0.485	0.788
	Pr	**0.958**	0.782	0.809	**0.999**	0.323	0.783	**0.995**	0.755	0.902
Log type: uprooted	Beta	0.448	-0.014	0.805	-0.408	-0.037	-0.143	0.019	**-1.539**	-0.085
	Pr	0.741	0.489	0.923	0.243	0.470	0.426	0.523	**0.044**	0.440
Decay stage	Beta	-0.388	-0.528	**-1.492**	-0.649	**-2.457**	**1.321**	-0.698	-1.397	**-4.132**
	Pr	0.348	0.162	**0.020**	0.136	**0.017**	**0.971**	0.150	0.214	**0.001**
Sequencing depth	Beta	**1.297**	-0.257	1.039	-0.352	**1.170**	**1.648**	-0.829	**1.653**	0.362
	Pr	**0.965**	0.339	0.936	0.333	**0.951**	**0.985**	0.156	**0.980**	0.682
Per-phylum species richness										
Asco-mycota	Beta	**-0.050**	-0.018	-0.004	0.010	0.006	-0.008	-0.008	0.015	0.015
	Pr	**0.021**	0.162	0.412	0.717	0.630	0.361	0.352	0.767	0.765
Basidio-mycota	Beta	-0.034	0.067	-0.036	-0.007	-0.033	0.035	**-0.100**	-0.030	-0.043
	Pr	0.264	0.937	0.192	0.424	0.187	0.814	**0.038**	0.275	0.186
Log type: felled	Beta	**1.041**	0.697	0.318	**1.568**	-0.388	0.608	**1.507**	0.368	0.530
	Pr	**0.959**	0.897	0.701	**0.996**	0.252	0.839	**0.967**	0.696	0.784
Log type: uprooted	Beta	0.315	-0.231	0.791	-0.404	-0.051	-0.158	0.129	**-1.434**	-0.015
	Pr	0.670	0.324	0.919	0.247	0.477	0.421	0.590	**0.049**	0.479
Decay stage	Beta	-0.340	**-0.947**	**-1.514**	-0.604	**-2.277**	**1.282**	-0.123	-1.057	**-4.151**
	Pr	0.353	**0.081**	**0.016**	0.157	**0.020**	**0.956**	0.454	0.291	**0.001**
Sequencing depth	Beta	**1.333**	-0.177	1.116	-0.393	**1.345**	**1.719**	-0.796	**1.628**	0.332
	Pr	**0.975**	0.389	0.946	0.31	**0.971**	**0.990**	0.154	**0.976**	0.687

Model and predictors		Inoculated species								
		Antrodia piceata	*Antrodiella citrinella*	*Fomitopsis rosea*	*Perenniporia subacida*	*Physisporinus crocatus*	*Postia guttulata*	*Skeletocutis odora*	*Skeletocutis stellae*	*Steccherinum collabens*
Community composition PA										
LV1	Beta	**-1.041**	-0.511	-0.389	**0.733**	-0.063	-0.329	-0.782	-0.424	-0.027
	Pr	**0.003**	0.113	0.159	**0.976**	0.423	0.261	0.062	0.139	0.469
LV2	Beta	-0.253	**-0.477**	**0.606**	**-0.964**	0.216	0.287	**-0.852**	**-1.214**	-0.628
	Pr	0.192	**0.044**	**0.985**	**0.026**	0.778	0.785	**0.005**	**0.027**	0.058
Log type: felled	Beta	0.075	-0.056	0.492	1.365	-0.238	-0.077	1.316	-1.053	0.103
	Pr	0.545	0.468	0.748	0.926	0.343	0.463	0.916	0.158	0.536
Log type: uprooted	Beta	0.410	0.493	**1.330**	-0.431	0.022	-0.071	-0.131	-1.187	0.017
	Pr	0.745	0.818	**0.974**	0.260	0.511	0.457	0.394	0.112	0.515
Decay stage	Beta	-0.521	-0.245	**-1.276**	-0.730	**-2.236**	**1.522**	-0.393	-0.703	**-4.842**
	Pr	0.262	0.353	**0.038**	0.158	**0.017**	**0.985**	0.305	0.364	**0.000**
Sequencing depth	Beta	**1.321**	-0.087	0.815	-0.210	**1.140**	**1.751**	-1.031	1.380	0.468
	Pr	**0.984**	0.462	0.885	0.403	**0.961**	**0.989**	0.108	0.933	0.738
DNA amount										
DNA amount	Beta	-0.112	0.022	-0.160	0.104	0.217	-0.099	**0.269**	**0.633**	-0.078
	Pr	0.230	0.574	0.083	0.802	0.907	0.233	**0.958**	**0.997**	0.282
Log type: felled	Beta	**1.014**	0.365	0.695	**1.732**	-0.370	0.485	**1.633**	0.454	0.753
	Pr	**0.974**	0.762	0.915	**0.999**	0.268	0.798	**0.983**	0.746	0.890
Log type: uprooted	Beta	0.575	-0.042	0.666	-0.376	-0.133	-0.179	0.118	**-1.861**	0.022
	Pr	0.817	0.469	0.874	0.245	0.424	0.420	0.580	**0.015**	0.514
Decay stage	Beta	-0.530	-0.558	**-1.252**	-0.660	**-2.368**	**1.442**	**-1.234**	-0.887	**-4.273**
	Pr	0.258	0.168	**0.029**	0.141	**0.023**	**0.984**	**0.023**	0.334	**<0.001**
Sequencing depth	Beta	**1.156**	-0.296	0.912	-0.246	**1.113**	**1.568**	-0.732	**1.782**	0.266
	Pr	**0.954**	0.303	0.918	0.349	**0.951**	**0.988**	0.171	**0.955**	0.627
Community composition RRA										
LV1	Beta	0.292	**0.888**	0.119	-0.340	-0.255	-0.122	-0.275	-0.358	0.379
	Pr	0.869	**1**	0.693	0.133	0.240	0.389	0.227	0.169	0.844
LV2	Beta	-0.295	-0.339	-0.341	-0.236	-0.268	0.030	0.084	0.075	-0.160
	Pr	0.108	0.201	0.133	0.239	0.194	0.541	0.597	0.574	0.329
Log type: felled	Beta	**0.974**	1.000	**1.220**	**1.651**	-0.135	0.210	**2.004**	0.586	1.203
	Pr	**0.965**	0.885	**0.976**	**0.999**	0.428	0.592	**0.994**	0.797	0.948
Log type: uprooted	Beta	0.130	0.055	0.815	-0.432	-0.188	-0.258	-0.022	-1.389	-0.131
	Pr	0.59	0.537	0.916	0.229	0.398	0.344	0.488	0.056	0.409
Decay stage	Beta	-0.533	-0.488	-1.035	-0.579	**-2.353**	**1.419**	**-1.139**	-1.000	**-4.291**
	Pr	0.289	0.218	0.07	0.192	**0.014**	**0.983**	**0.025**	0.282	**0.000**
Sequencing depth	Beta	**1.184**	-0.153	0.818	-0.357	1.207	**1.727**	-0.256	**1.630**	0.497
	Pr	**0.972**	0.399	0.896	0.313	0.948	**0.991**	0.371	**0.977**	0.758

Table S6. Estimated regression parameters (Beta) and their posterior probabilities (*Pr(Beta>0* and *Pr(Beta<0*) for different variants of the ten most common species model separately for each inoculated species. The models include either the presence-absence or relative read abundance of the ten most common OTUs in the resident community, and variable selection either was or was not applied. In addition, each model includes the environmental abiotic predictors log type (*LogT* with levels felled (*F*) and uprooted (*U*) and broken log type included as an intercept), decay stage (*DecayS*), and sequencing depth (*SeqDepth*). Beta parameters describe how the target species respond to each predictor. In models without variable selection, the posterior probability for a negative response is not shown separately as for those models Pr(Beta<0)=1-Pr(Beta>0). For models with variable selection, Pr(Beta=0) may be non-zero and hence, the posterior probabilities are shown both for both positive and negative responses. Positive (negative) responses to predictors with at least 0.95 (0.05 or less) posterior probability are indicated with darker red (blue), and responses with at least 0.90 (0.10 or less) posterior probability are indicated with lighter red (blue). Taxonomic assignments for the OTUs are shown in Table S7.

		Presence-absence					Relative read abundance				
		no variable selection		*with variable selection*			*no variable selection*		*with variable selection*		
	Predictor	Beta	Pr (Beta>0)	Beta	Pr (Beta>0)	Pr (Beta<0)	Beta	Pr (Beta>0)	Beta	Pr (Beta>0)	Pr (Beta<0)
Antrodia piceata	OTU01	-0.305	0.316	-0.347	0	0.48	0.036	0.497	-0.023	0.01	0.021
	OTU02	-0.230	0.377	-0.333	0	0.481	**-5.140**	**0.04**	-0.605	0.005	0.133
	OTU04	-0.711	0.144	-1.644	0	0.935	-3.418	0.115	-0.289	0.011	0.077
	OTU32	-0.705	0.146	-0.564	0.001	0.603	2.129	0.803	-0.031	0.005	0.023
	OTU03	-0.835	0.141	-1.511	0.001	0.85	-6.566	0.104	-0.616	0.006	0.092
	OTU26	-0.568	0.198	-0.572	0.003	0.603	1.783	0.779	0.009	0.018	0.015
	OTU38	**-1.368**	**0.044**	-1.041	0	0.705	0.014	0.472	-4.1E-04	0.01	0.015
	OTU56	**1.139**	**0.954**	-0.084	0.001	0.164	0.227	0.565	0.012	0.018	0.012
	OTU05	-0.044	0.488	-0.445	0.003	0.494	**-3.709**	**0.02**	-0.137	0.008	0.06
	OTU07	-1.165	0.075	**-2.513**	**0**	**0.956**	-4.443	0.077	-0.161	0.003	0.047
	LogT: F	0.376	0.677	0.875	0.793	0.207	-0.284	0.398	0.801	0.896	0.104
	LogT: U	0.569	0.755	1.507	0.936	0.064	-0.348	0.324	0.308	0.7	0.3
	DecayS	-0.497	0.337	-0.246	0.412	0.588	-1.025	0.258	-0.468	0.294	0.706
	SeqDep	1.375	0.948	**1.929**	**0.987**	**0.013**	**1.573**	**0.964**	**1.201**	**0.959**	**0.041**
Antrodiella citrinella	OTU01	-0.239	0.308	-0.319	0.002	0.527	-0.133	0.466	-0.009	0.005	0.015
	OTU02	0.373	0.775	-0.131	0.003	0.289	1.759	0.827	0.030	0.019	0.007
	OTU05	0.839	0.946	-0.094	0.002	0.184	4.174	0.921	0.478	0.101	0.007
	OTU18	-0.099	0.406	-0.197	0.004	0.375	0.661	0.687	0.003	0.01	0.012
	OTU38	-0.657	0.19	-0.258	0.004	0.401	1.717	0.794	0.072	0.033	0.009
	OTU121	0.033	0.522	-0.147	0.003	0.28	-2.884	0.052	-0.041	0.007	0.028
	OTU04	0.369	0.753	-0.141	0	0.275	0.601	0.642	-4.1E-06	0.014	0.011
	OTU12	0.312	0.736	-0.144	0.002	0.26	4.065	0.911	0.139	0.035	0.004
	OTU32	0.144	0.608	-0.150	0.002	0.275	1.433	0.894	0.017	0.017	0.009
	OTU40	0.430	0.731	-0.125	0.005	0.216	2.347	0.779	0.037	0.022	0.007
	LogT: F	0.287	0.662	0.281	0.666	0.334	0.493	0.701	0.335	0.735	0.265
	LogT: U	-0.262	0.347	-0.115	0.401	0.599	0.312	0.698	-0.013	0.497	0.503
	DecayS	**-1.280**	**0.038**	-0.752	0.128	0.872	-0.971	0.12	-0.551	0.18	0.82
	SeqDep	-0.053	0.474	-0.388	0.235	0.765	0.080	0.561	-0.233	0.34	0.66
Fomitopsis rosea	OTU01	-4.516	0.272	-0.424	0	0.533	-1.344	0.265	-0.020	0.009	0.019
	OTU02	-0.444	0.704	-0.275	0	0.409	1.708	0.691	-0.030	0.006	0.02
	OTU13	0.446	0.79	-0.198	0.004	0.326	4.297	0.837	0.066	0.028	0.01
	OTU56	0.523	0.315	-0.471	0.002	0.521	0.984	0.7	0.050	0.039	0.007
	OTU05	-0.357	0.729	-0.151	0	0.268	-1.151	0.361	-0.022	0.008	0.016
	OTU32	0.429	0.226	-0.673	0	0.676	**-3.690**	**0.044**	-0.023	0.012	0.015
	OTU40	-0.487	0.54	-0.227	0.006	0.337	**-6.299**	**0.012**	-0.192	0.003	0.065
	OTU04	0.041	0.334	-0.325	0.001	0.418	-1.588	0.298	-0.037	0.011	0.022
	OTU18	**-0.377**	**0.013**	-1.258	0	0.915	**-5.156**	**0.005**	-0.415	0.006	0.125
	OTU26	**-1.358**	**0.001**	**-2.089**	**0**	**0.971**	**-4.802**	**0.003**	-0.718	0.004	0.22
	LogT: F	-1.860	0.788	0.695	0.797	0.203	-0.038	0.486	0.690	0.903	0.097
	LogT: U	**0.721**	**0.993**	**1.699**	**0.972**	**0.028**	**1.972**	**0.986**	0.946	0.932	0.068
	DecayS	1.804	0.104	**-2.269**	**0.025**	**0.975**	**-2.790**	**0.022**	**-1.178**	**0.045**	**0.955**
	SeqDep	-1.285	0.744	0.732	0.782	0.218	-0.329	0.388	0.579	0.802	0.198

		Presence-absence					Relative read abundance				
		no variable selection		*with variable selection*			*no variable selection*		*with variable selection*		
		Beta	Pr (Beta>0)	Beta	Pr (Beta>0)	Pr (Beta<0)	Beta	Pr (Beta>0)	Beta	Pr (Beta>0)	Pr (Beta<0)
Perenniporia subacida	OTU02	-0.086	0.459	-0.163	0	0.288	**-7.809**	**0.022**	-0.042	0.005	0.023
	OTU01	1.130	0.944	-0.089	0.003	0.308	1.998	0.814	0.027	0.023	0.007
	OTU56	0.714	0.862	-0.120	0.002	0.187	-0.784	0.281	-0.003	0.008	0.013
	OTU04	-0.234	0.391	-0.247	0.002	0.246	-1.542	0.238	-0.056	0.008	0.029
	OTU03	0.168	0.586	-0.138	0.002	0.381	**9.012**	**0.989**	0.046	0.017	0.006
	OTU18	-0.355	0.311	-0.191	0.001	0.252	-2.245	0.096	-0.027	0.01	0.028
	OTU66	-0.344	0.31	-0.359	0	0.324	3.385	0.891	0.031	0.025	0.01
	OTU06	0.139	0.594	-0.185	0.001	0.478	**4.130**	**0.953**	0.107	0.042	0.006
	OTU25	-1.061	0.075	-0.547	0.002	0.315	0.830	0.614	0.015	0.016	0.011
	OTU05	0.880	0.906	-0.141	0.001	0.604	**4.183**	**0.979**	0.042	0.032	0.014
	LogT: F	**1.537**	**0.967**	**1.326**	**0.981**	**0.241**	**1.501**	**0.952**	**1.494**	**0.996**	**0.004**
	LogT: U	0.043	0.518	-0.506	0.231	0.019	-0.178	0.417	-0.384	0.257	0.743
	DecayS	-0.901	0.148	-1.087	0.079	0.769	0.186	0.554	-0.631	0.154	0.846
	SeqDep	0.239	0.59	-0.310	0.349	0.921	-0.429	0.335	-0.194	0.411	0.589
Physisporinus crocatus	OTU02	0.755	0.852	-0.014	0	0.026	0.436	0.548	0.028	0.018	0.008
	OTU01	-0.434	0.296	-0.041	0	0.039	-0.030	0.498	0.010	0.011	0.008
	OTU18	**-1.575**	**0.023**	-3.605	0	0.881	**-7.595**	**0.004**	-1.291	0.008	0.216
	OTU04	-1.341	0.056	-0.163	0	0.107	-2.148	0.209	0.002	0.015	0.015
	OTU15	-0.012	0.48	-0.012	0	0.016	-0.480	0.385	-0.015	0.01	0.019
	OTU03	0.696	0.754	-0.031	0	0.031	7.267	0.921	0.040	0.019	0.006
	OTU26	0.101	0.54	-0.015	0	0.022	-0.877	0.354	-0.016	0.007	0.019
	OTU32	0.108	0.579	-0.032	0	0.039	0.458	0.589	-0.005	0.011	0.016
	OTU56	-0.746	0.169	-0.976	0	0.392	-0.886	0.257	-0.022	0.01	0.027
	OTU40	-0.546	0.253	-0.948	0	0.351	0.184	0.513	0.016	0.015	0.016
	LogT: F	-0.094	0.455	-1.300	0.162	0.838	-0.609	0.231	-0.315	0.327	0.673
	LogT: U	-0.026	0.499	0.833	0.721	0.279	0.110	0.561	0.077	0.543	0.457
	DecayS	**-2.814**	**0.03**	**-288.075**	**0**	**1**	**-2.566**	**0.026**	**-2.371**	**0.014**	**0.986**
	SeqDep	**2.073**	**0.991**	**4.160**	**0.994**	**0.006**	**1.919**	**0.992**	**1.243**	**0.969**	**0.031**
Postia guttulata	OTU02	-1.229	0.051	-0.846	0	0.797	-1.581	0.288	-0.024	0.013	0.021
	OTU01	0.532	0.761	-0.298	0.001	0.461	-1.595	0.238	-0.015	0.011	0.018
	OTU26	-0.410	0.284	-0.678	0	0.706	-2.613	0.069	-0.018	0.007	0.019
	OTU04	0.074	0.57	-0.439	0.004	0.53	0.633	0.642	0.002	0.015	0.011
	OTU40	-0.191	0.428	-0.228	0.004	0.354	**-10.780**	**0.011**	-0.087	0.009	0.033
	OTU38	-0.089	0.465	-0.241	0.002	0.353	0.307	0.533	0.011	0.019	0.019
	OTU56	**1.506**	**0.963**	-0.096	0.002	0.184	**3.788**	**0.97**	0.095	0.047	0.012
	OTU36	-0.156	0.427	-0.626	0.002	0.616	-0.648	0.354	-0.008	0.014	0.02
	OTU92	0.335	0.627	-0.198	0.003	0.307	3.627	0.778	0.010	0.016	0.017
	OTU95	0.359	0.625	-0.199	0.002	0.295	3.459	0.855	0.110	0.044	0.01
	LogT: F	-0.145	0.441	0.204	0.598	0.402	-0.412	0.339	0.335	0.72	0.28
	LogT: U	-0.051	0.504	-0.345	0.306	0.694	0.372	0.682	-0.254	0.362	0.638
	DecayS	**2.051**	**0.995**	**1.751**	**0.992**	**0.008**	**2.092**	**0.985**	**1.395**	**0.98**	**0.02**
	SeqDep	**2.059**	**0.978**	1.237	0.929	0.071	**2.238**	**0.981**	**1.606**	**0.987**	**0.013**

		Presence-absence					Relative read abundance				
		no variable selection		*with variable selection*			*no variable selection*		*with variable selection*		
		Beta	Pr (Beta>0)	Beta	Pr (Beta>0)	Pr (Beta<0)	Beta	Pr (Beta>0)	Beta	Pr (Beta>0)	Pr (Beta<0)
Skeletocutis odora	OTU01	0.175	0.59	-0.258	0.002	0.42	3.005	0.905	0.020	0.018	0.008
	OTU02	-0.290	0.346	-0.236	0.001	0.404	**-12.369**	**0.002**	-0.672	0.002	0.124
	OTU04	-0.068	0.459	-0.262	0.003	0.413	-1.784	0.279	-0.194	0.006	0.069
	OTU05	0.107	0.576	-0.299	0	0.422	-0.333	0.468	-0.044	0.016	0.026
	OTU66	-0.066	0.455	-0.450	0.001	0.537	2.643	0.747	0.014	0.02	0.014
	OTU26	0.096	0.557	-0.216	0.001	0.373	0.079	0.509	0.007	0.015	0.011
	OTU32	0.336	0.677	-0.164	0.003	0.3	0.885	0.611	0.009	0.018	0.008
	OTU03	-0.667	0.179	-0.395	0.002	0.51	**-10.807**	**0.011**	-1.616	0.005	0.225
	OTU25	-0.186	0.391	-0.352	0.003	0.482	-6.074	0.093	-0.210	0.01	0.051
	OTU37	-0.995	0.077	-0.808	0.002	0.751	-8.141	0.066	-0.687	0.011	0.112
	LogT: F	**2.055**	**0.989**	**1.488**	**0.978**	**0.022**	1.316	0.872	**1.572**	**0.97**	**0.03**
	LogT: U	0.155	0.594	0.316	0.712	0.288	0.838	0.834	0.177	0.635	0.365
	DecayS	-1.032	0.136	**-1.176**	**0.038**	**0.962**	-0.259	0.438	-0.969	0.078	0.922
	SeqDep	-0.683	0.222	-0.595	0.224	0.776	-0.871	0.235	-0.491	0.262	0.738
Skeletocutis stellae	OTU02	0.600	0.771	-0.002	0	0.006	2.676	0.779	0.037	0.024	0.007
	OTU01	-0.171	0.425	-0.010	0	0.016	-2.606	0.112	-0.012	0.021	0.022
	OTU03	-1.143	0.122	**-124.491**	**0**	**1**	-4.727	0.231	-0.339	0.006	0.053
	OTU04	-1.433	0.066	**-141.210**	**0**	**1**	-3.106	0.18	-0.246	0.01	0.064
	OTU05	-0.275	0.365	-0.030	0	0.034	-3.292	0.21	-0.070	0.01	0.025
	OTU18	0.257	0.59	-0.008	0	0.012	0.813	0.62	0.011	0.025	0.015
	OTU56	-0.081	0.453	-0.007	0	0.008	0.744	0.683	-0.009	0.017	0.017
	OTU66	0.040	0.515	-0.045	0	0.045	0.290	0.559	-0.005	0.011	0.014
	OTU06	-0.249	0.39	-0.090	0	0.068	0.688	0.589	-0.005	0.014	0.015
	OTU26	-0.668	0.189	-0.154	0.001	0.117	**-4.286**	**0.027**	-0.130	0.006	0.042
	LogT: F	0.260	0.616	0.091	0.52	0.48	-0.225	0.398	0.431	0.743	0.257
	LogT: U	-0.882	0.21	**-82.853**	**0.025**	**0.975**	-1.198	0.109	-1.381	0.062	0.938
	DecayS	-0.762	0.365	-38.964	0.419	0.581	-0.412	0.417	-1.133	0.244	0.756
	SeqDep	**2.326**	**0.973**	**2.346**	**0.986**	**0.014**	**2.798**	**0.986**	**1.697**	**0.984**	**0.016**
Steccherinum collabens	OTU01	0.085	0.553	-0.020	0	0.025	2.776	0.78	-0.011	0.013	0.017
	OTU02	0.728	0.787	-0.007	0	0.011	**10.196**	**0.983**	0.136	0.04	0.016
	OTU18	-0.058	0.465	-0.022	0	0.03	-4.436	0.057	-0.056	0.006	0.03
	OTU15	**2.297**	**0.988**	-0.002	0	0.006	**6.712**	**0.998**	0.552	0.15	0.006
	OTU03	**-2.337**	**0.008**	**-23.461**	**0**	**0.958**	**-17.817**	**0.007**	-2.509	0.005	0.246
	OTU38	-0.656	0.264	-0.057	0	0.046	-2.314	0.268	-0.005	0.013	0.014
	OTU04	0.434	0.678	-0.010	0	0.015	-1.979	0.328	0.019	0.016	0.009
	OTU05	**1.743**	**0.971**	-0.004	0	0.013	**9.972**	**0.962**	0.899	0.132	0.003
	OTU26	-0.890	0.16	-0.016	0	0.021	**-6.026**	**0.031**	-0.058	0.006	0.031
	OTU56	0.367	0.661	-0.008	0	0.009	2.020	0.709	0.051	0.027	0.01
	LogT: F	0.029	0.515	-22.359	0.055	0.945	0.331	0.608	0.413	0.717	0.283
	LogT: U	-0.929	0.172	-0.364	0.324	0.676	0.415	0.627	-0.150	0.422	0.578
	DecayS	**-6.552**	**0**	**-486.158**	**0**	**1**	**-6.124**	**0.005**	**-4.477**	**0**	**1**
	SeqDep	0.475	0.644	-0.162	0.458	0.542	0.617	0.677	0.382	0.705	0.295

Table S7. Taxonomic assignment for the ten most common resident OTUs in Table S6 for each target species. Taxonomically unidentified clusters at each rank were assigned to unique placeholder names starting with *pseudo*. Orders and families listed as *incertae sedis* refer to cases where the order of family is not described but some lower ranks are.

OTU	Phylum	Class	Order	Family	Genus	Species
OTU001	*Ascomycota*	*Leotiomycetes*	*Helotiales*	*Xenopolyscytalum* incertae sedis	*Xenopolyscytalum*	*Xenopolyscytalum pinea*
OTU002	*Ascomycota*	*Leotiomycetes*	*Helotiales*	*Tympanidaceae*	*Collophora*	pseudospecies 0320
OTU003	*Ascomycota*	*Eurotiomycetes*	*Chaetothyriales*	*Herpotrichiellaceae*	*Exophiala*	pseudospecies 0486
OTU004	*Ascomycota*	*Sordariomycetes*	*Coniochaetales*	*Coniochaetaceae*	pseudogenus 0050	pseudospecies 1175
OTU005	*Ascomycota*	*Eurotiomycetes*	*Chaetothyriales*	*Herpotrichiellaceae*	*Cladophialophora*	pseudospecies 0198
OTU006	*Ascomycota*	*Sordariomycetes*	*Lulworthiales*	*Lulworthiaceae*	*Zalerion*	*Zalerion arboricola*
OTU007	*Ascomycota*	*Leotiomycetes*	*Helotiales*	*Infundichalara* incertae sedis	*Infundichalara*	pseudospecies 0658
OTU012	*Ascomycota*	*Dothideomycetes*	*Capnodiales*	*Cladosporiaceae*	*Cladosporium*	*Cladosporium grevilleae*
OTU013	*Ascomycota*	*Eurotiomycetes*	*Chaetothyriales*	*Herpotrichiellaceae*	*Cladophialophora*	pseudospecies 0223
OTU015	*Basidiomycota*	*Agaricomycetes*	*Auriculariales*	*Auriculariaceae*	*Auricularia*	pseudospecies 0061
OTU018	*Basidiomycota*	*Agaricomycetes*	*Polyporales*	*Fomitopsidaceae*	*Fomitopsis*	*Fomitopsis pinicola*
OTU025	*Ascomycota*	*Leotiomycetes*	*Helotiales*	*Vibrisseaceae*	*Phialocephala*	*Phialocephala scopiformis*
OTU026	*Ascomycota*	*Leotiomycetes*	*Helotiaceae*	*Helotiaceae*	*Ascocoryne*	*Ascocoryne cylichnium*
OTU032	*Ascomycota*	*Leotiomycetes*	*Helotiales*	*Tympanidaceae*	*Collophora*	pseudospecies 0321
OTU036	*Basidiomycota*	*Agaricomycetes*	*Russulales*	*Bondarzewiaceae*	*Heterobasidion*	*Heterobasidion annosum*
OTU037	*Ascomycota*	*Dothideomycetes*	*Capnodiales*	pseudofamily 0075	pseudogenus 0440	pseudospecies 1740
OTU038	*Ascomycota*	pseudoclass 0001	pseudoorder 0291	pseudofamily 0786	pseudogenus 1225	pseudospecies 2932
OTU040	*Ascomycota*	*Saccharomycetes*	*Saccharomycetales*	*Saccharomycetaceae*	*Kuraishia*	*Kuraishia capsulata*
OTU056	*Ascomycota*	*Sordariomycetes*	*Microascales*	*Endoconidiophora* incertae sedis	*Endoconidiophora*	*Endoconidiophora polonica*
OTU066	*Ascomycota*	*Saccharomycetes*	*Saccharomycetales*	*Saccharomycetaceae*	pseudogenus 0197	pseudospecies 1413
OTU092	*Basidiomycota*	*Microbotryomycetes*	*Trigonosporomyces* incertae sedis	*Trigonosporomyces* incertae sedis	*Trigonosporomyces*	pseudospecies 1090
OTU095	*Ascomycota*	*Saccharomycetes*	*Saccharomycetales*	*Saccharomycetaceae*	*Kuraishia*	*Kuraishia molischiana*
OTU121	*Ascomycota*	*Saccharomycetes*	*Danielozyma* incertae sedis	*Danielozyma* incertae sedis	pseudogenus 0296	pseudospecies 1549

References

Abarenkov, K. 2022. sh_matching_data_0_5_v3.zip. Supporting files for EOSC-Nordic service (SH matching analysis v2.0.0). URL: https://app.plutof.ut.ee/filerepository/view/5582954

Abarenkov, K., P. Somervuo, R. H. Nilsson, P. M. Kirk, T. Huotari, N. Abrego, and O. Ovaskainen. 2018. Protax-fungi: a web-based tool for probabilistic taxonomic placement of fungal internal transcribed spacer sequences. New Phytologist 220:517–525.

Callahan, B. J. 2020. DADA2 ITS Pipeline Workflow (1.8) [WWW Document]. URL https://benjjneb.github.io/dada2/ITS_workflow.html (accessed 18 Sep 2020).

Callahan, B. J., P. J. McMurdie, M. J. Rosen, A. W. Han, A. J. A. Johnson, and S. P. Holmes. 2016. DADA2: High-resolution sample inference from Illumina amplicon data. Nature Methods 13:581–583.

Edgar, R. C. 2010. Search and clustering orders of magnitude faster than BLAST. Bioinformatics 26: 2460–2461. doi:10.1093/bioinformatics/btq461

Gardes, M., and T. D. Bruns. 1993. ITS primers with enhanced specificity for basidiomycetes – application to the identification of mycorrhizae and rusts. Molecular Ecology 2:113–118.

Hyvärinen, E., A. Juslén, E. Kemppainen, A. Uddström, and U.-M. Liukko, editors. 2019. The 2019 Red List of Finnish Species. Ympäristöministeriö & Suomen ympäristökeskus, Helsinki.

Justo, A., O. Miettinen, D. Floudas, B. Ortiz-Santana, E. Sjökvist, D. Lindner, K. Nakasone, T. Niemelä, K.-H. Larsson, L. Ryvarden, and D. S. Hibbett. 2017. A revised family-level classification of the Polyporales (Basidiomycota). Fungal Biology 121: 798-824. doi:10.1016/j.funbio.2017.05.010

Kotiranta, H., K. Junninen, P. Halme, I. Kytövuori, T. von Bonsdorff, T. Niskanen, and K. Liimatainen. 2019. Aphyllophoroid fungi. Pages 234–247 in E. Hyvärinen, A. Juslén, E. Kemppainen, A. Uddström, and U.-M. Liukko, editors. The 2019 Red List of Finnish Species. Ministry of the Environment & Finnish Environment Institute, Helsinki.

Kotiranta, H., K. Junninen, R. Saarenoksa, J. Kinnunen, and I. Kytövuori. 2010. Aphyllophorales & Heterobasidiomycetes. Pages 249–263 in P. Rassi, E. Hyvärinen, A. Juslén, and I. Mannerkoski, editors. The 2010 Red Lish of Finnish Species. Ministry of the Environment & Finnish Environment Institute, Helsinki.

Landau, W. M. 2021. The targets R package: a dynamic Make-like function-oriented pipeline toolkit for reproducibility and high-performance computing. Journal of Open Source Software 6: 2959. doi: 10.21105/joss.02959

Martin, M. 2011. Cutadapt removes adapter sequences from high-throughput sequencing reads. EMBnet.journal, 17(1): 10–12. doi:10.14806/ej.17.1.200

Natural Resources Institute Finland 2019. Monilähteisen valtakunnan metsien inventoinnin (MVMI) kartta-aineisto 2019. https://kartta.luke.fi/index.html (accessed 19 Apr 2021)

Niemelä, T. 2016. Suomen käävät. Finnish Museum of Natural History LUOMUS, Helsinki, Finland.

Nordén, J., N. Abrego, L. Boddy, C. Bässler, A. Dahlberg, P. Halme, M. Hällfors, S. Maurice, A. Menkis, O. Miettinen, R. Mäkipää, O. Ovaskainen, R. Penttilä, S. Saine, T. Snäll, and K. Junninen. 2020. Ten principles for conservation translocations of threatened wood- inhabiting fungi. Fungal Ecology 44:1–9.

Ovaskainen, O., N. Abrego, P. Somervuo, I. Palorinne, B. Hardwick, J. M. Pitkänen, N. R. Andrew, P. A. Niklaus, N. M. Schmidt, S. Seibold, J. Vogt, E. V. Zakharov, P. D. N. Hebert, T. Roslin, and N. V. Ivanova. 2020. Monitoring Fungal Communities With the Global Spore Sampling Project. Frontiers in Ecology and Evolution 7:1–9.

R Core Team. 2022. R: A language and environment for statistical computing. R Foundation for Statistical Computing, Vienna, Austria. https://www.R-project.org/

Renvall, P. 1995. Community structure and dynamics of wood-rotting Basidiomycetes on decomposing conifer trunks in northern Finland. Karstenia 35:1–51.

Rognes, T., T. Flouri, B. Nichols, C. Quince, and F. Mahé. 2016. VSEARCH: a versatile open source tool for metagenomics. PeerJ 4:e2584.

Vu, D., R. H. Nilsson, and G. J. M. Verkley. 2022. Dnabarcoder: An open-source software package for analysing and predicting DNA sequence similarity cutoffs for fungal sequence identification. Molecular Ecology Resources 22. doi:10.1111/1755-0998.13651.

White, T. J., T. D., Bruns, S. B. Lee, and J. W. Taylor. 1990. Amplification and direct sequencing of fungal ribosomal RNA genes for phylogenetics. Pages 315–322 in M. A. Innis, D. H. Gelfand, J. J. Sninsky, and T. J. White, editors. PCR protocols: a guide to methods and applications. Academic Press, New York.
